# The population genetics characteristics of a 90 locus panel of microhaplotypes

**DOI:** 10.1007/s00439-021-02382-0

**Published:** 2021-10-13

**Authors:** Andrew J. Pakstis, Neeru Gandotra, William C. Speed, Michael Murtha, Curt Scharfe, Kenneth K. Kidd

**Affiliations:** Department of Genetics, Yale University School of Medicine, New Haven, CT 06520 USA

## Abstract

Single-nucleotide polymorphisms (SNPs) and small genomic regions with multiple SNPs (microhaplotypes, MHs) are rapidly emerging as novel forensic investigative tools to assist in individual identification, kinship analyses, ancestry inference, and deconvolution of DNA mixtures. Here, we analyzed information for 90 microhaplotype loci in 4009 individuals from 79 world populations in 6 major biogeographic regions. The study included multiplex microhaplotype sequencing (mMHseq) data analyzed for 524 individuals from 16 populations and genotype data for 3485 individuals from 63 populations curated from public repositories. Analyses of the 79 populations revealed excellent characteristics for this 90-plex MH panel for various forensic applications achieving an overall average effective number of allele values (A_e_) of 4.55 (range 1.04–19.27) for individualization and mixture deconvolution. Population-specific random match probabilities ranged from a low of 10^–115^ to a maximum of 10^–66^. Mean informativeness (I_n_) for ancestry inference was 0.355 (range 0.117–0.883). 65 novel SNPs were detected in 39 of the MHs using mMHseq. Of the 3018 different microhaplotype alleles identified, 1337 occurred at frequencies > 5% in at least one of the populations studied. The 90-plex MH panel enables effective differentiation of population groupings for major biogeographic regions as well as delineation of distinct subgroupings within regions. Open-source, web-based software is available to support validation of this technology for forensic case work analysis and to tailor MH analysis for specific geographical regions.

**Supplementary Information:**

The online version contains supplementary material available at 10.1007/s00439-021-02382-0.

## Introduction

For many years, the DNA markers for forensic practice have been short tandem repeat (STR) loci that are highly polymorphic with different numbers of repeat units at each locus (Budowle et al. 1998). Over the years, the numbers of standard STR loci have increased and the similarities of the different commercial panels and those in different countries have increased (Butler and Hill 2012; Schumm et al. 2013; Guo et al. 2014; Novroski et al. 2019). Other types of markers have been proposed starting with SNPs especially in the early 2000s (cf., Pakstis et al. 2007 for early studies). Early forensic studies of SNPs were focused on individual identification (Sanchez et al. 2006; Pakstis et al. 2007), on panels of SNPs for inferring population ancestry (e.g., Shriver et al. 2004; Tishkoff and Kidd 2004; Phillips et al. 2007), and on SNPs for phenotype (e.g., Lamason et al. 2005; Walsh et al. 2011; Walsh et al. 2013). Several commercial panels of SNPs have been introduced, some of which combine SNPs with STRs, for analysis using Massively Parallel Sequencing (MPS). MPS has also allowed the further development of a new type of genetic marker, microhaplotypes (Kidd et al. 2013,2014).

Microhaplotypes (microhaplotypes, MHs) have been defined as small genomic regions of less than ~ 300 bp with two or more polymorphisms, usually SNPs, resulting in at least three common haplotypes in the population (cf., review in Oldoni et al. 2019). They were first proposed as potentially highly informative and useful genetic markers for forensics, anthropology, and biomedical research generally. Their desirable characteristics include multiple alleles with high heterozygosity and low mutation rates. Since then, MHs have been studied by many researchers with clear demonstration of their potential for forensic, medical, and anthropologic applications (Bulbul et al. 2018; Chen et al. 2018; Kidd et al. 2018a,^2021^; Cheung et al. 2019; Phillips et al. 2019; Puente et al. 2020a; Puente et al. 2020b), but they have not yet been incorporated into routine forensic casework.

Although conceived of for use with MPS, the original studies which were designed to evaluate the potential for microhaplotypes (Kidd et al. 2013,2014) used TaqMan to type individual SNPs and then PHASE (Stephens et al. 2001) to determine the genotypes and haplotype frequencies. The SNPs that were chosen to study were those of at least modest frequency (5–10%) in some populations, those that were not in complete LD with another, and those for which TaqMan assays were available. Other factors could be included in selection of SNPs if different ultimate objectives were favored (Kidd and Speed 2015; Kidd et al. 2018a). While MPS was not used in these exploratory studies, it was clear that the existence of MPS was what made study of microhaplotypes relevant. Now, there have been several studies that have used MPS successfully to study panels of microhaplotypes on multiple individuals and/or populations (Turchi et al. 2019; Chen et al. 2018; Oldoni et al. 2019; Bennett et al. 2019; Gandotra et al. 2020; Puente et al. 2020a; Puente et al. 2020b; Kureshi et al. 2020; Wu et al. 2021a; Wu et al. 2021b).

We previously presented a panel of 90 microhaplotypes evaluated using data for 26 populations extracted from the 1000 Genomes (1 KG) project data (1000 Genomes Consortium Project 2015) as well as data on 155 individuals from four other populations studied using multiplex microhaplotype sequencing (mMHseq) of all 90 loci (Gandotra et al. 2020). The 90 loci had a high overall effective number of alleles (A_e_) in the 30 populations studied (average A_e_ > 5.08). Analyses of frequency variation among populations showed that some of the loci had significant variation among populations. To be of maximal value in forensics as well as in other areas of research, a panel of loci needs a broad set of reference population frequencies. To that end, we have now assembled and analyzed sequence-based data on 4009 individuals in 79 populations for these 90 microhaps. These results also demonstrate the value of microhaplotypes for biomedical and anthropologic studies of human populations.

## Materials and methods

### Population samples

The 4009 individuals in 79 populations studied (Table 1) include 524 individuals in 16 populations that we have typed by MPS (Table 2). The DNA for the individuals sequenced was purified using phenol–chloroform from lymphoblastic cell lines that are part of the Kidd Lab collection. Greater detail of the population samples can be found in ALFRED (alfred.med.yale.edu). Comparable data for 3485 other individuals in 63 populations that were curated from public repositories: the Human Genome Diversity Project (HGDP) which includes individuals sequenced from the Kidd Lab collection of world population samples (see Table 2 and Bergstrom et al. 2020); the Genome Asia database (Genome Asia100 K Consortium 2019); and the 1000 Genomes (1000 Genomes Consortium Project et al. 2015).

**Table 1:** The 79 populations

World Region	Sample Size (*N*)	Population	Abbrev	Source
Central Africa	46	Biaka	BIA	KL, HGDP
West Africa	113	Gambians	GWD	1 KG
	22	Mandenka	MDK	HGDP
	85	Mende	MSL	1 KG
	99	Esan	ESN	1 KG
	22	Yoruba, Benin City	YOR	HGDP
	108	Yoruba, Ibadan	YRI	1 KG
East Africa	99	Luhya, Kenya	LWK	1 KG
	28	Zaramo	ZRM	KL
	45	Chagga	CGA	KL
	20	Masai, Tanzania	MAS	KL
	17	Masai, Kenya	MKK	GA
	40	Sandawe	SND	KL
	96	Afro-Caribbeans	ACB	1 KG
	61	AfrAmer, Southwest USA	ASW	1 KG
	31	Ethiopian Jews	ETJ	KL
North Africa	27	Mozabites	MZB	HGDP
Southwest Asia	46	Bedouin	BDN	HGDP
	42	Druze	DRZ	HGDP
	46	Palestinians	PLS	HGDP
	42	Adygei	ADY	KL, HGDP
Europe	28	Sardinians	SRD	HGDP
	107	Tuscans	TSI	1 KG
	107	Iberians	IBS	1 KG
	23	Basques, France	BSQ	HGDP
	15	Orcadians	ORC	HGDP
	99	CEPH Europeans	CEU	1 KG
	91	Great Britain	GBR	1 KG
	28	French	FRN	HGDP
	25	Russians	RUS	HGDP
	99	Finns	FIN	1 KG
So Central Asia	22	Kalash, Pakistan	KLS	HGDP
	35	Pathans, Pakistan	PTH	HGDP, GA
	20	Gujjar, Pakistan	GJJ	GA
	24	Balochi, Pakistan	BLC	HGDP
	24	Sindhi, Pakistan	SNH	HGDP
	25	Makrani, Pakistan	MKR	HGDP
	25	Brahui, Pakistan	BRH	HGDP
	24	Burusho, Pakistan	BRS	HGDP
	96	Punjabi, Lahore	PJL	1 KG
	103	Gujarati	GIH	1 KG
	34	Urban Chennai	CNI	GA
	34	Urban Bangalore	BGL	GA
	102	Telugu	ITU	1 KG
	102	Tamil, SriLanka	STU	1 KG
	86	Bengali, Bangladesh	BEB	1 KG
	17	Lambada, India	LMB	GA
	17	Agharia, India	AGH	GA
	19	Mahar, India	MHR	GA
	20	Toda, India	TOD	GA
	15	Oraon, India	ORA	GA
	17	KondaReddy, India	KND	GA
	20	Birhor, India	BIR	GA
	26	Hazara, Pakistan	HZR	HGDP, GA
East Asia	45	Khanty	KTY	KL
	20	Mog, India	MOG	GA
	87	Buryat	BUR	GA
	48	Yakut	YAK	KL
	150	Koreans	KRE	GA
	104	Japanese, Tokyo	JPT	1 KG
	30	Japanese, Tokyo healthy controls	JPA	GA
	27	Japanese	JPH	HGDP
	103	HanChinese, Beijing	CHB	1 KG
	43	HanChinese, HGDP	HAN	HGDP
	105	SouthernHanChinese	CHS	1 KG
	93	Dai	CDX	1 KG
	99	Vietnamese	KHV	1 KG
	23	Cambodians	CBD	KL
	36	Atayal	ATL	KL
Oceania	25	Austronesians, Indonesia	ASN	GA
	21	Ati, Philippines	ATI	GA
	20	Flores, Rampasasa, Indonesia	FLR	GA
	29	Aeta, Philippines	AET	GA
	37	Micronesians	MCR	KL
	30	Papuans, NewGuinea	PNG	KL, HGDP
	23	Nasioi	NAS	KL
Americas	35	Pima, Mexico	PMM	KL
	27	Maya	MAY	KL, HGDP
	85	Peruvians	PEL	1 KG

Abbreviations in Source column–1 KG   Thousand Genomes Consortium, *GA*  Genome Asia Project, *HGDP* CEPH’s Human Genome Diversity Project, *KL*  Kidd Lab. Approximately one-third of the HGDP population samples derive from Kidd Lab population samples

**Table 2:** Summary of individuals from Kidd lab populations included in analyses after sequencing and passing QC

	Kidd lab population sample	Individuals analyzed (*N*)			
		Sequenced, passed QC		Sequenced data from HGDP^b^	Total analyzed
		This study	Gandotra et al. 2020		
1	Biaka	32	4	10	46
2	Masai	20	0		20
3	Sandawe	0	40		40
4	Zaramo	0	28		28
5	Chagga	0	45		45
6	Ethiopian Jews	31	0		31
7	Adygei	0	30	12	42
8	Khanty	45	0		45
9	Yakut	48	0		48
10	Atayal	36	0		36
11	Cambodians	23	0		23
12	Nasioi	23	0		23
13	Papuans, New Guinea	22	0	8	30
14	Micronesians	37	0		37
15	Pima, Mexico	35	0		35
16	Maya	25	0	2	27
17	Southern Tunisians^a^	4	0		0
18	EuroAmericans^a^	0	6		0
19	Chinese, Taiwan^a^	0	2		0
	Totals	381	155	32	556

Some Kidd lab population sample individuals are also noted that are included in the analyses but sequenced via HGDP study

^a^Groups not included in population genetics analyses because of the small numbers of individuals with data

^b^See (Bergstrom et al. 2020). The individuals from the HGDP are from the same Kidd lab population samples

The 536 sequenced individuals included 155 individuals sequenced previously (Gandotra et al. 2020) and 381 individuals that were sequenced and passed quality control steps in this study (Table 2). Twelve individuals were also successfully sequenced from other groups including 4 samples from Southern Tunisia in this study and 6 Euro-Americans and 2 Chinese from Taiwan in Gandotra et al. (2020). These were excluded from statistical analyses, because the sample sizes were too small. They will be included in future studies as more sequenced samples accumulate. The data from all samples sequenced are available on the Scharfe lab mMHseq website (see Data Availability).

### Data collection

The descriptions of the 90 microhaplotype loci and the primers for MPS are described in Gandotra et al. (2020) (cf. Table S2 in that paper) as are the detailed mMHseq methods. Table 3 provides an overview of key characteristics of the 90 microhaps. The mMHseq libraries of 48 individuals and two non-template water controls were sequenced in a single Illumina MiSeq run for all 90 microhaplotypes. This number of samples per run assures that each sample receives sufficient sequence read coverage based on the assay’s empirically established performance parameters. Data analysis included sample demultiplexing, primer trimming, read alignment to the human reference genome (hg19/GRCh37), data quality control (QC), DNA variant calling (GATK UnifiedGenotyper (GATK UG), and SNP phasing for each microhaplotype using Read backed phasing tools from GATK to phase the SNP’s along the microhaplotype (McKenna et al. 2010). Following identification of variants at each of the 90 MH loci in the 536 individuals using mMHseq, base calls at the same variant sites were extracted for 3485 individuals from various whole-genome sequencing (WGS) repositories.

**Table 3:** Microhaplotype list sorted by chromosome and initial SNP in upstream nt-position (build GRCh37)

Cnt	Microhaplotype	Chr	Nt position for initial SNP upstream side	Molecular extent base pairs	Total 79-population SNP count	Avg A_e_ 79 population	Rosenberg I_n_ 79 population
1	mh01KK-172	1	1,486,834	226	8	3.29	0.354
2	mh01KK-001	1	3,743,109	283	11	3.62	0.464
3	mh01KK-205	1	18,722,692	242	9	3.94	0.150
4	mh01KK-212^a^	1	202,616,547	243	17	9.71	0.883
5	mh01KK-117	1	204,633,340	189	9	4.22	0.303
6	mh01NK-001	1	230,820,351	280	5	3.16	0.205
7	mh01KK-213^a^	1	232,811,740	216	16	4.78	0.393
8	mh02KK-022^a^	2	3,172,438	249	9	5.37	0.436
9	mh02KK-138	2	46,191,983	249	8	2.94	0.292
10	mh02KK-029^a^	2	69,138,957	236	14	5.41	0.354
11	mh02KK-013^a^	2	105,833,031	221	9	3.65	0.313
12	mh02KK-031^a^	2	123,395,790	252	14	4.15	0.328
13	mh02KK-134	2	161,079,411	104	8	4.87	0.344
14	mh02KK-136	2	228,092,334	198	7	4.74	0.236
15	mh02KK-014^a^	2	228,524,072	239	16	8.96	0.601
16	mh02KK-015^a^	2	240,004,773	221	11	4.38	0.433
17	mh03KK-016^a^	3	14,377,432	201	12	3.11	0.220
18	mh03KK-017^a^	3	37,516,028	179	7	4.37	0.286
19	mh03KK-047^a^	3	45,166,218	243	7	3.66	0.214
20	mh03KK-018^a^	3	117,156,240	224	13	4.75	0.588
21	mh03KK-150	3	131,645,972	185	9	3.30	0.117
22	mh04KK-010	4	1,986,720	261	8	2.73	0.172
23	mh04KK-030	4	3,666,211	284	9	4.07	0.617
24	mh04KK-013	4	68,444,102	201	8	3.73	0.250
25	mh05KK-169^a^	5	1,898,501	234	7	4.53	0.303
26	mh05KK-170	5	2,447,910	256	14	9.75	0.812
27	mh05KK-020	5	38,881,438	199	7	3.55	0.164
28	mh05KK-178^a^	5	67,309,764	231	9	4.69	0.295
29	mh06KK-090^a^	6	29,937,692	280	17	4.73	0.347
30	mh06KK-104^a^	6	165,798,851	188	5	4.26	0.342
31	mh06KK-008	6	169,656,029	275	14	4.81	0.661
32	mh07KK-009^a^	7	18,861,121	182	16	6.82	0.525
33	mh08KK-039	8	3,516,789	228	18	4.36	0.484
34	mh08KK-131^a^	8	5,461,399	227	15	3.91	0.262
35	mh08KK-137^a^	8	31,083,232	195	12	7.38	0.575
36	mh09KK-161	9	344,087	289	10	2.99	0.558
37	mh09KK-010^a^	9	2,288,476	264	10	4.46	0.450
38	mh09KK-145^a^	9	4,763,309	218	9	5.08	0.388
39	mh09KK-153	9	103,969,642	247	7	5.69	0.521
40	mh09KK-157	9	135,862,478	155	7	3.53	0.237
41	mh10KK-162^a^	10	3,160,652	266	13	5.18	0.356
42	mh10KK-167^a^	10	12,545,332	222	8	4.73	0.275
43	mh10KK-170	10	78,910,042	190	7	2.61	0.288
44	mh11KK-180	11	1,690,714	271	12	5.10	0.567
45	mh11KK-181^a^	11	2,819,168	128	10	4.27	0.302
46	mh11KK-183^a^	11	20,020,042	217	12	6.43	0.438
47	mh11KK-190^a^	11	97,176,319	224	7	4.55	0.301
48	mh11KK-191	11	99,880,163	190	7	3.52	0.288
49	mh12KK-199^a^	12	12,229,744	209	8	4.03	0.172
50	mh12KK-201^a^	12	27,800,327	177	15	8.36	0.804
51	mh12KK-202	12	30,170,229	154	5	3.07	0.137
52	mh12KK-046	12	118,889,488	289	8	4.85	0.256
53	mh12KK-209^a^	12	130,308,483	191	7	4.52	0.255
54	mh13KK-213	13	23,765,409	273	11	5.09	0.339
55	mh13KK-215	13	36,451,857	242	10	4.40	0.322
56	mh13KK-217	13	46,865,888	235	10	5.04	0.387
57	mh13KK-218	13	54,060,710	263	7	7.62	0.481
58	mh13KK-225	13	66,712,622	207	7	3.43	0.231
59	mh13KK-221^a^	13	101,759,088	253	12	6.39	0.681
60	mh13KK-222^a^	13	106,642,644	252	13	4.56	0.382
61	mh13KK-223	13	110,806,689	237	11	4.22	0.256
62	mh14KK-227^a^	14	52,334,089	215	10	4.53	0.297
63	mh14KK-048	14	74,250,537	194	8	3.22	0.283
64	mh15KK-067	15	46,870,730	196	7	3.26	0.317
65	mh15KK-066	15	52,484,819	271	10	3.39	0.259
66	mh16KK-049	16	7,209,185	250	19	4.64	0.410
67	mh16KK-302	16	7,587,615	233	10	3.04	0.296
68	mh16KK-255	16	81,970,352	193	14	3.63	0.342
69	mh16KK-259^a^	16	83,973,819	248	14	7.85	0.571
70	mh16KK-011^a^	16	84,285,727	198	11	5.43	0.407
71	mh16KK-262^a^	16	87,669,318	258	13	4.67	0.330
72	mh17KK-272	17	52,942,335	260	11	3.74	0.203
73	mh17KK-012^a^	17	77,141,265	245	13	3.09	0.275
74	mh17KK-013^a^	17	77,276,404	245	10	3.58	0.233
75	mh17KK-278^a^	17	78,761,546	187	7	5.48	0.439
76	mh18KK-293	18	76,089,732	237	7	3.46	0.324
77	mh19KK-299	19	22,729,500	182	10	4.05	0.281
78	mh19KK-300^a^	19	51,451,043	182	7	4.25	0.349
79	mh20KK-306^a^	20	895,313	219	7	4.89	0.346
80	mh20KK-307	20	16,513,215	208	8	3.66	0.237
81	mh20KK-058	20	48,844,260	247	9	2.82	0.198
82	mh21KK-315	21	21,880,086	184	7	4.46	0.214
83	mh21KK-316	21	27,782,968	255	7	3.36	0.258
84	mh21KK-318^a^	21	41,260,129	235	10	4.05	0.291
85	mh21KK-320	21	43,062,859	271	10	4.95	0.279
86	mh21KK-313	21	43,942,101	207	8	2.49	0.278
87	mh21KK-324	21	46,714,536	179	9	4.74	0.404
88	mh22KK-328^a^	22	18,518,651	244	7	3.92	0.264
89	mh22KK-061	22	44,763,550	217	10	3.52	0.172
90	mh22KK-340^a^	22	49,060,976	261	11	5.66	0.422

Total 79-population SNP count is the total number of different SNPs in the specific locus haplotypes across all 79 populations

^a^Indicates microhaplotypes that are not in the ALFRED allele frequency database

### Data analyses

Effective number of alleles (A_e_) is a measure that standardizes the information among diverse populations for their different frequencies among the multiple alleles (Kimura and Crow 1964; Kidd and Speed 2015). A_e_ for a locus is calculated as the inverse of homozygosity, A_e_ = 1/sum(p_i_^2^). As such, it is the number of equally frequent alleles that would yield the same heterozygosity as the observed set of alleles with diverse frequencies. This measure is good for evaluating multiallelic loci (such as microhaplotypes) for individualization and mixture analysis. Informativeness (I_n_) for measuring allele frequency differences among populations was calculated according to Rosenberg et al. (2003). This measure is appropriate for evaluating loci for their ability to infer population ancestry of an individual and relationships among populations.

For the extracted data that were not phased in the respective repositories, the haplotypes were inferred using PHASE version 2.1.1 (Stephens et al. 2001; Stephens and Scheet 2005). For all of the QC passed samples, the phasing was obtained directly from the reads for each of the MH loci.

### Structure, PCA, and population trees

To help visualize clustering of individuals in populations, we used version 2.3.4 of the STRUCTURE software (Pritchard et al. 2000). The program was run under the standard admixture model assuming correlated allele frequencies. The input data consisted of the microhaplotype genotype profiles for all individuals in the 79 populations. The program was run 20 times at each K level from *K* = 2 to *K* = 16 with 10,000 burn-in and 10,000 Markov Chain Monte Carlo (MCMC) iterations. The result with the highest likelihood of the 20 runs was selected to illustrate the results for a given *K* value.

To help visualize clustering of populations, we used Principal Component Analyses (PCA). We used the XLSTAT 2019 software (http://www.xlstat.com/en/about-us/company.html) on the matrix of haplotype allele frequencies for all 90 microhaplotype loci in the populations relevant to each analysis.

We also conducted tree analyses for the 79 populations using pairwise Tau genetic distances (Kidd and Cavalli-Sforza 1974) and methods and logic described in Kidd and Sgaramella-Zonta (1971) and Cherni et al. (2016). Analyses started with the Neighbor Joining tree (Saitou and Nei 1987), which gives an approximate Least Squares fit, and then explored similar tree structures by an exact Least Squares fit to the defining set of linear equations. The Neighbor Joining (NJ) program employed is part of the PHYLIP software package (Felsenstein 1989,2009). The Drawtree utility (version 3.69) in the PHYLIP package was used to plot the postscript images of the best population trees.

## Results

### mMHseq data analysis and quality control

Assay performance was assessed using our algorithms for monitoring sequence read coverage on three levels: samples, amplicons (loci), and sequence bases (Fig. S1, Table S2). Any sample that failed this QC was removed from further analysis. The first QC metric (sample coverage), defined as the number of reads per sample, was used for detecting samples that failed in the multiplex PCR. An average read depth across 384 samples was 705,536 reads per sample. Eight out of 384 samples had lower read depth coverage of less than 150,000 reads and were flagged for further analysis of amplicon and base coverage (Table S2 and Fig. S1). The second QC metric (amplicon coverage) was used to identify samples with partially failed amplification, such as individual amplicons that may have been insufficiently covered despite an overall normal read count for that sample. For each sample, we obtained the number of amplicons that had > 0.2-fold the mean amplicon coverage and used a threshold of 2 standard deviations below the mean to flag samples for review. This metric identified 4 samples with poor amplicon uniformity (Table S2 and Fig. S1). The third QC metric (base pairs) assessed base coverage for each sample, reasoning that if base coverage was sufficiently high, even samples with lower amplicon uniformity could be analyzed further. Five samples had a lower base coverage (< 75% of bases with 100 × reads per nucleotide per amplicon). Three samples failed QC at all three levels and were removed from the analysis, while the other samples flagged in one of the three QC steps yielded interpretable results in sequence analysis. Thus, final analyses are based on data for 381 individuals (Tables 2 and S2). Additionally, we investigated the data for MH genotypes that could have been due to allele dropouts. We found 4 MH alleles that were present only as homozygous MH genotypes in a single individual (but in different sequenced individuals for each allele type) and the inferred two alleles were the only occurrences of those alleles in the whole dataset; so, these genotypes were removed from the analyses.

We estimate that each genotype call was based, on average, by 7067 reads. That number is the average of the sequencing reads per locus (amplicon) in the last five sequencing runs, each of which involved sequencing of 48 individuals. Thus, sequencing of a total of 240 individuals contributed to this number. These are the right-most 62% of the reads in supplemental Fig. S1. Some variation in read numbers occurred among the five runs considered, but the variation in reads per locus was consistent; the distribution of the number of loci by the number of reads is given in Fig. S2. We note that except for 13 loci, there were more than 500 reads per allele per locus per individual. Only one locus, mh01KK-001, averaged fewer than 100 reads per allele with 75.3 reads per allele. In general, coverage per locus exceeds the clinical exome sequencing standard of 80×. It is unclear whether the differences in reads per locus per individual are inherent to the locus or are inherent just to the sequence or concentration of the specific primer pair used for the sequencing. A future effort will be made to better balance across loci to assure a higher minimum number of reads for all loci.

In summary, the mMHseq 90-plex data for the sequenced individuals from 16 populations are available at the Scharfe lab mMHseq website and have also been deposited in the Zenodo archive (see Data Availability). Our previous study (Gandotra et al. 2020) identified 717 SNPs in the 90 MHs for 30 populations, while this study of 79 populations recorded 905 SNPs in the 90 MHs (Table S1), which included 65 novel SNPs in 39 of the 90 MHs.

### Characteristics of MH markers

**Fig. 1:**
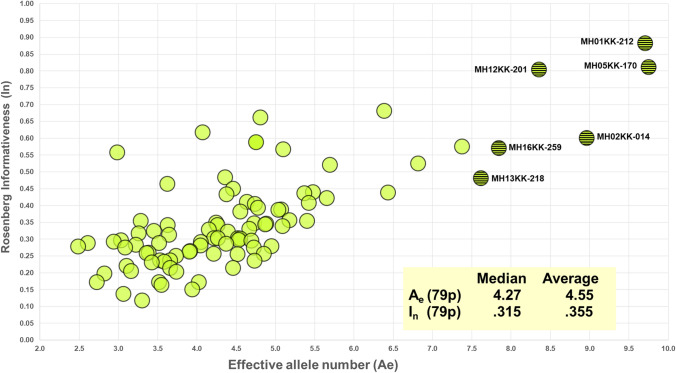
Scatterplot of 90 microhaplotypes by I_n_ and average A_e_ for 79 populations (79p). 6 MHs with highest A_e_ values in all 6 biogeograhic regions (cf Table 4) are shown as patterned circles

**Fig. 2:**
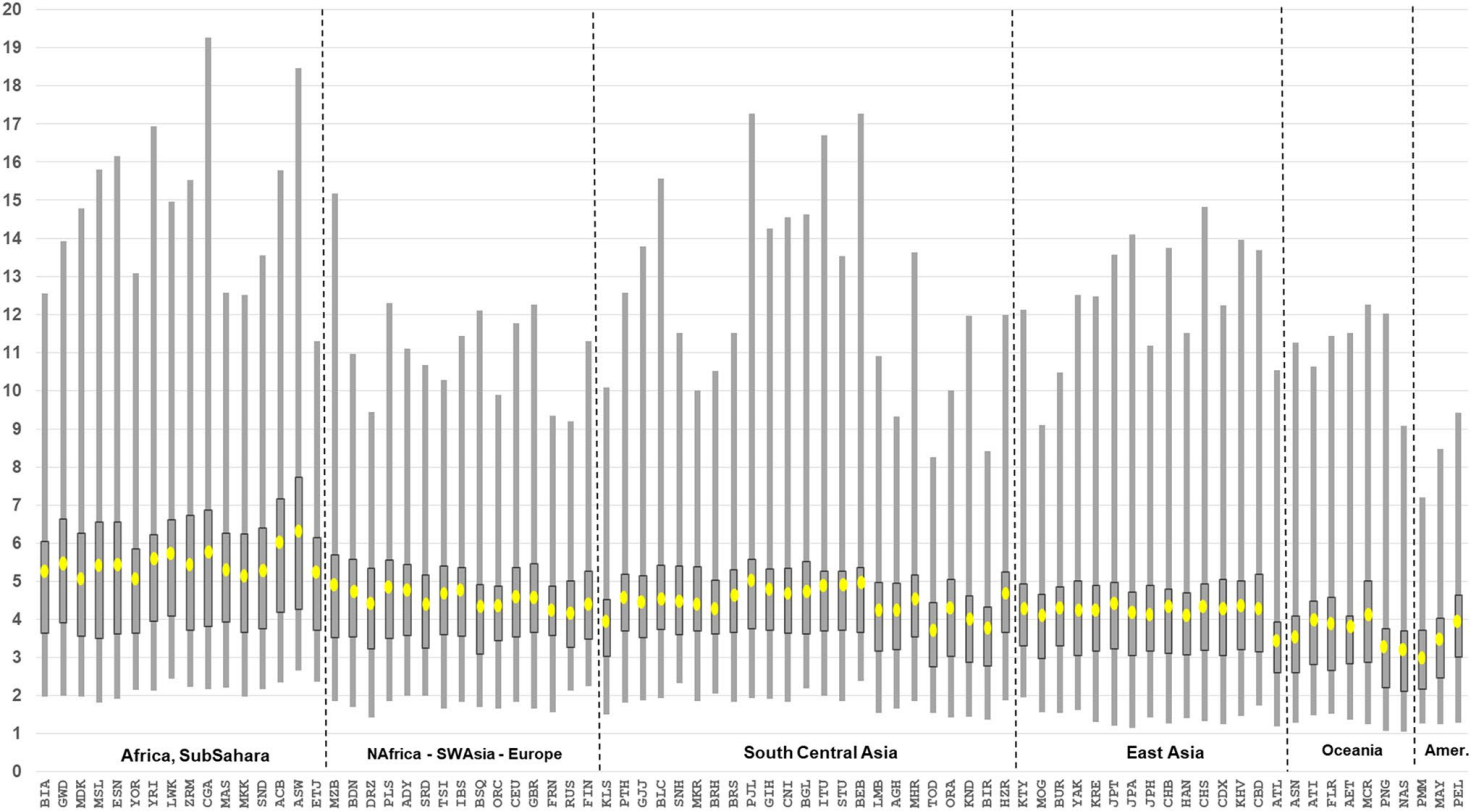
Box plots of A_e_ values for 90 microhaplotypes in each population. Box boundaries are at the 25^th^ and 75th percentiles; the light dot in the box marks average A_e_; the “whiskers” line extends from the minimum to maximun A_e_

**Fig. 3:**
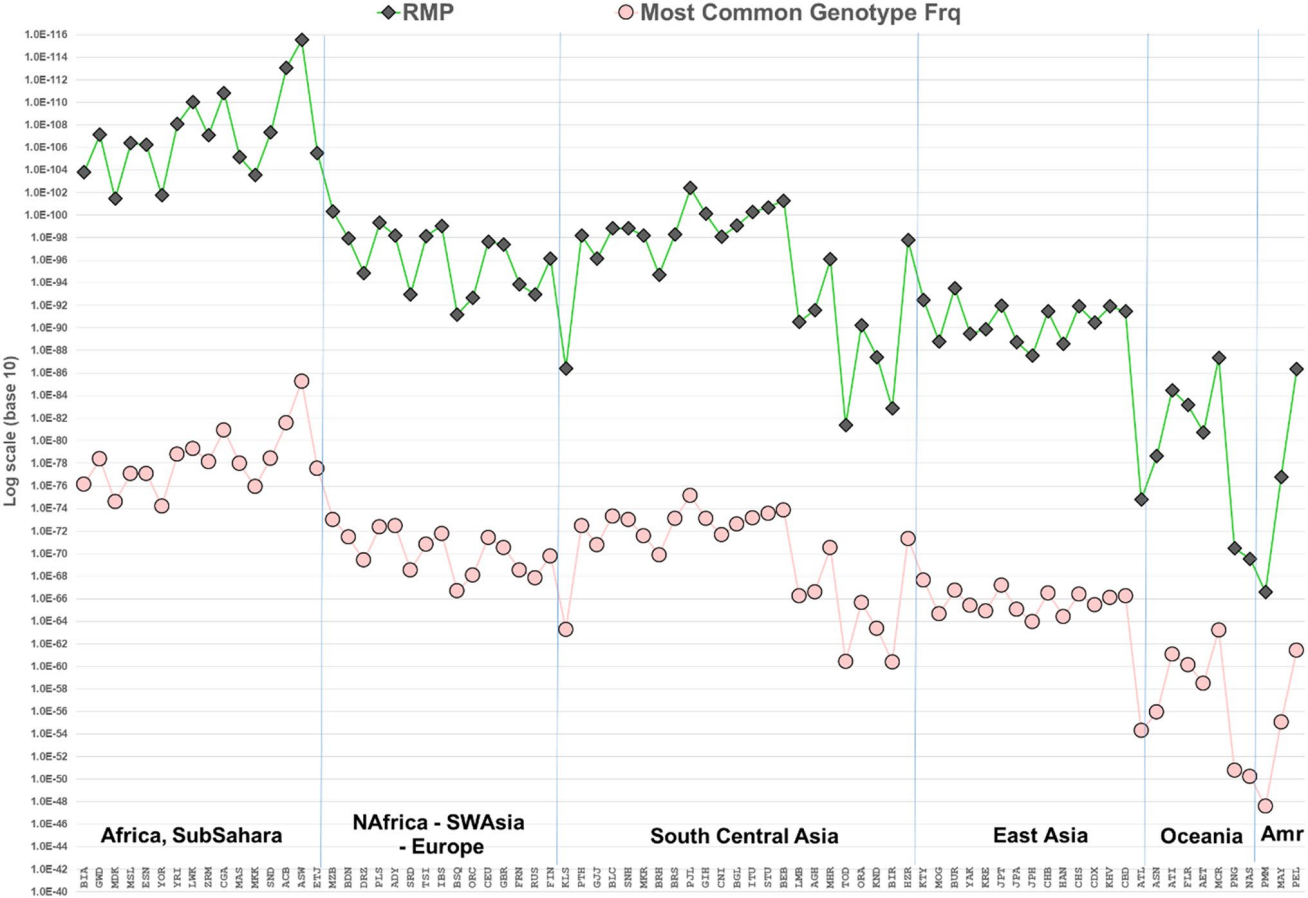
Random match probability and most common genotype frequency

As noted earlier, two statistics characterize the information in the markers with respect to variation within populations (A_e_) and variation among populations (I_n_). Figure 1 is a scatterplot of all 90 MHs according to I_n_ by average A_e_ for the total of 79 populations. Some of the markers rank very high by both criteria. The six MHs that are highest for A_e_ are shaded and included in Table 4. The clinal decrease in the average A_e_ across loci for populations that are farther from Africa is evident in Fig. 2. The markers have high heterozygosity with mean values of A_e_ ranging from 3.0 to more than 6.0 (Fig. 2) depending on the population. Among the 7110 individual population values (79 × 90) for A_e_, it is noteworthy that 81.7% are ≥ 3.0 and 96.8% are ≥ 2.0. Supplemental Fig. S3 plots the average A_e_ value for each of the 90 microhaplotypes. The most common genotype frequency in each population is also plotted in Fig. 3. Note that the specific genotype will likely be different in each population, the point being that no genotype is common anywhere when all 90 loci are considered.

**Table 4:** Region-specific average A_e_ for 6 highest ranking microhaplotypes worldwide

	Africa, Sub-Sahara	N Africa, SW Asia, Europe	South Central Asia	East Asia	Oceania	Americas
Number of populations:	16	15	24	14	7	3
Microhaplotype	Avg A_e_	Avg A_e_	Avg A_e_	Avg A_e_	Avg A_e_	Avg A_e_
mh01KK-212	10.35	9.26	9.03	11.88	9.29	5.98
mh02KK-014	14.06	8.00	7.93	7.89	6.88	5.79
mh05KK-170	9.16	9.53	11.60	9.33	7.23	9.81
mh12KK-201	11.28	7.76	7.94	8.11	6.13	7.07
mh13KK-218	7.61	7.95	8.35	7.79	4.33	8.84
mh16KK-259	6.87	6.52	7.49	10.24	9.75	8.35

The high A_e_ for many loci individually and on average across all populations indicates considerable variation within populations. A forensic measure, Random Match Probability (RMP), at a single locus is the sum over all the possible genotypes in the population of the squares of the genotype frequencies. In other words, it is the expected frequency (probability) for the population of, having randomly selected one individual, another unrelated individual will have that same specific genotype. For multi-locus genotypes, RMP becomes the product of the individual locus probabilities. It is often used in criminal cases to note how unlikely it is that someone else has the same genotype as a defendant. The RMP values are quite small for these 90 MH loci. However, RMP is population-specific and has a dramatic difference of 50 orders of magnitude depending on the population (Fig. 3). The range goes from the very small RMP values for Africans up to the much larger, but still highly probative, values for the Pacific and Native American populations: 10^–115^ up to 10^–66^. Globally, the probability of two unrelated individuals having the same genotype for these markers is vanishingly small. Note, this RMP is not the same as the probability that a random person will have the same genotype as a specific evidentiary genotype profile.

**Fig. 4:**
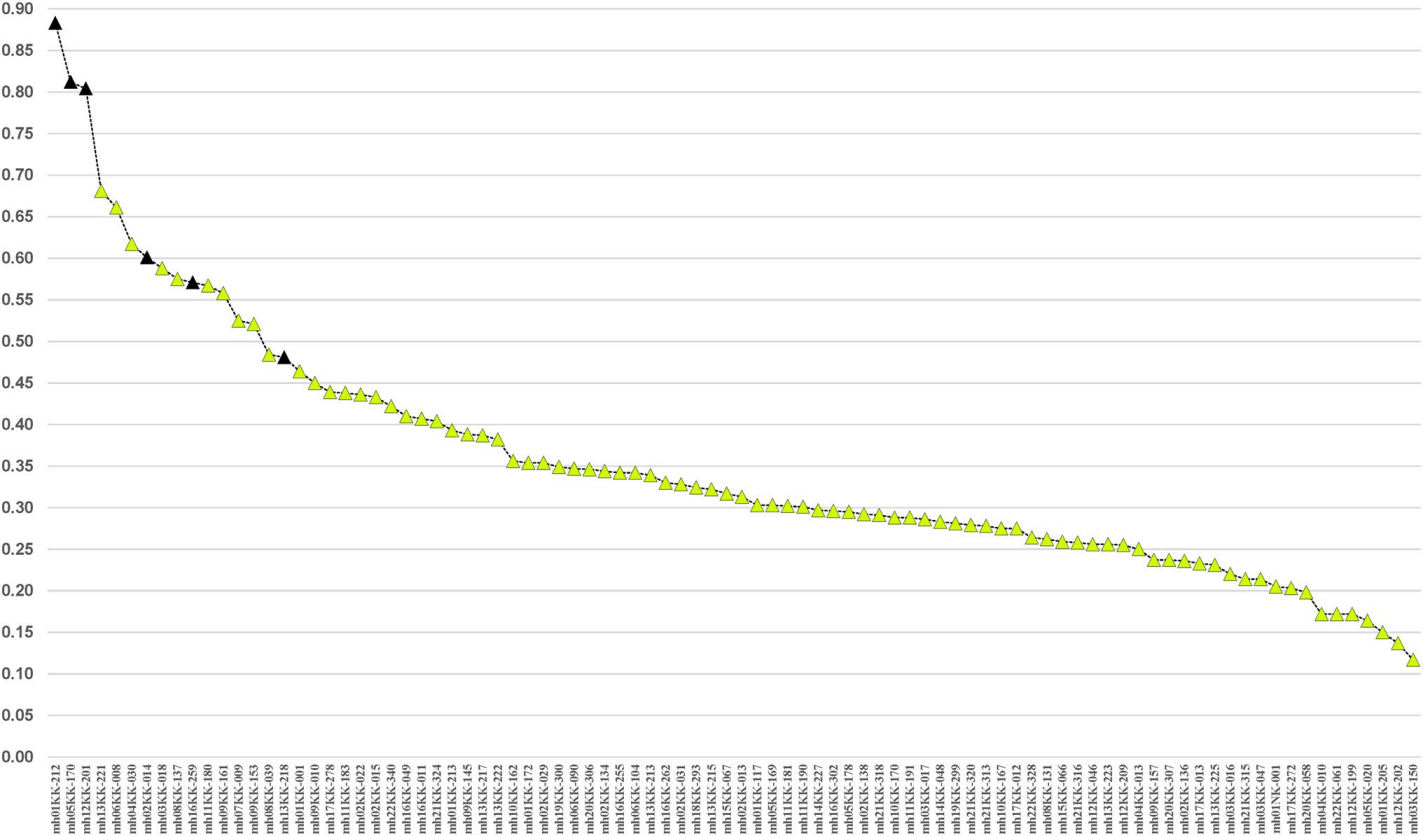
Rosenberg informativeness (I_n_) across 79 populations for each of 90 microhaplotypes. The 6 dark triangles correspond to the 6 MH with the highest Ae values in Fig. 1

Informativeness (I_n_) across the 79 population samples likewise shows considerable variation by locus (Fig. 4). The specific loci with the highest I_n_ values are clearly distinct in Fig. 1 as are those loci with the lowest I_n_ values.

### Inference of population relationships

#### Structure

**Fig. 5:**
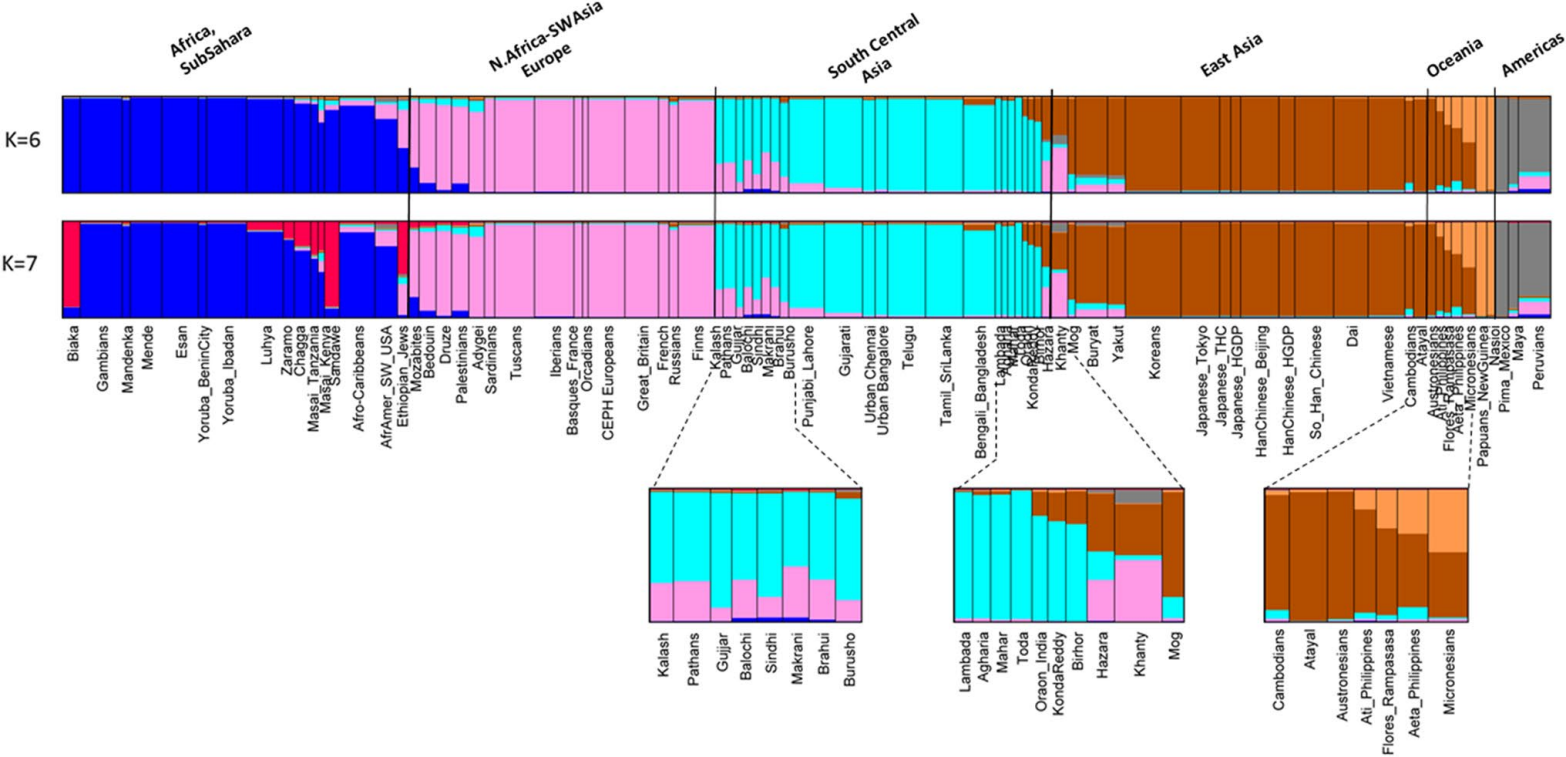
STRUCTURE population average bar plot at *K* = 6 and 7 for all 79 populations

**Fig. 6:**
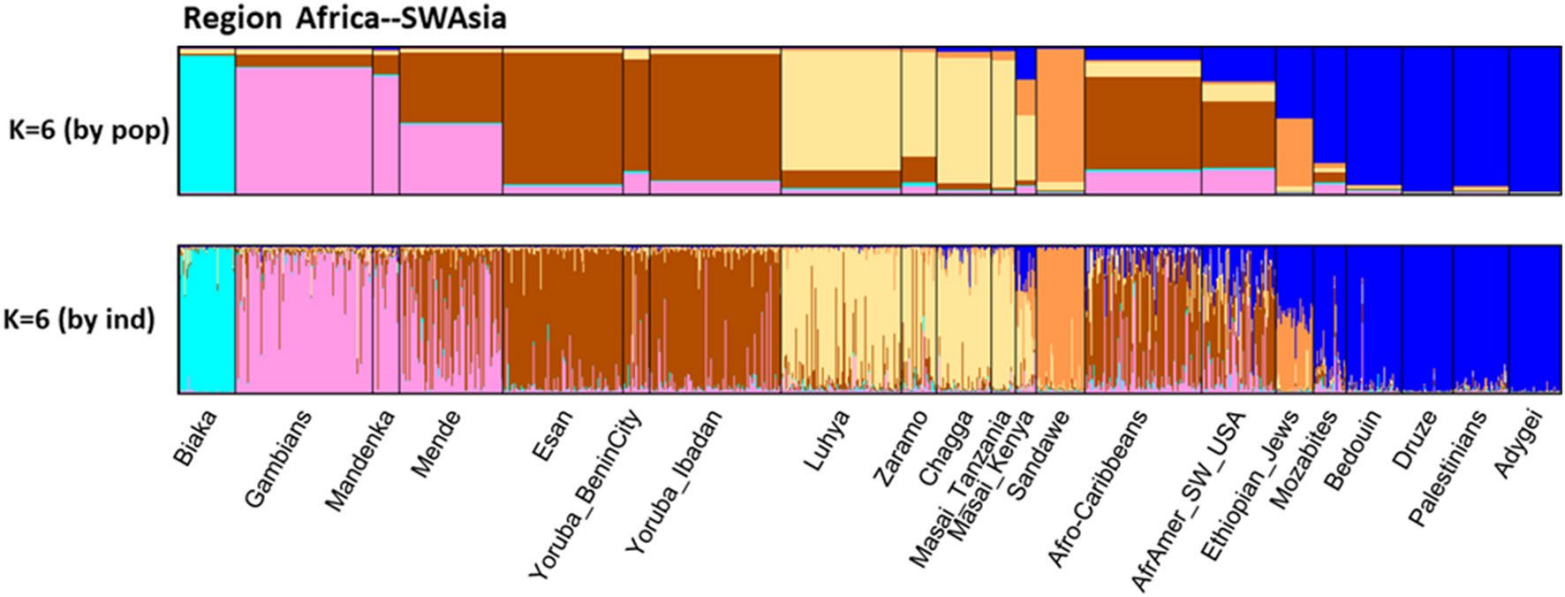
STRUCTURE of 21 populations from sub-Saharan Africa to Southwest Asia

**Fig. 7:**
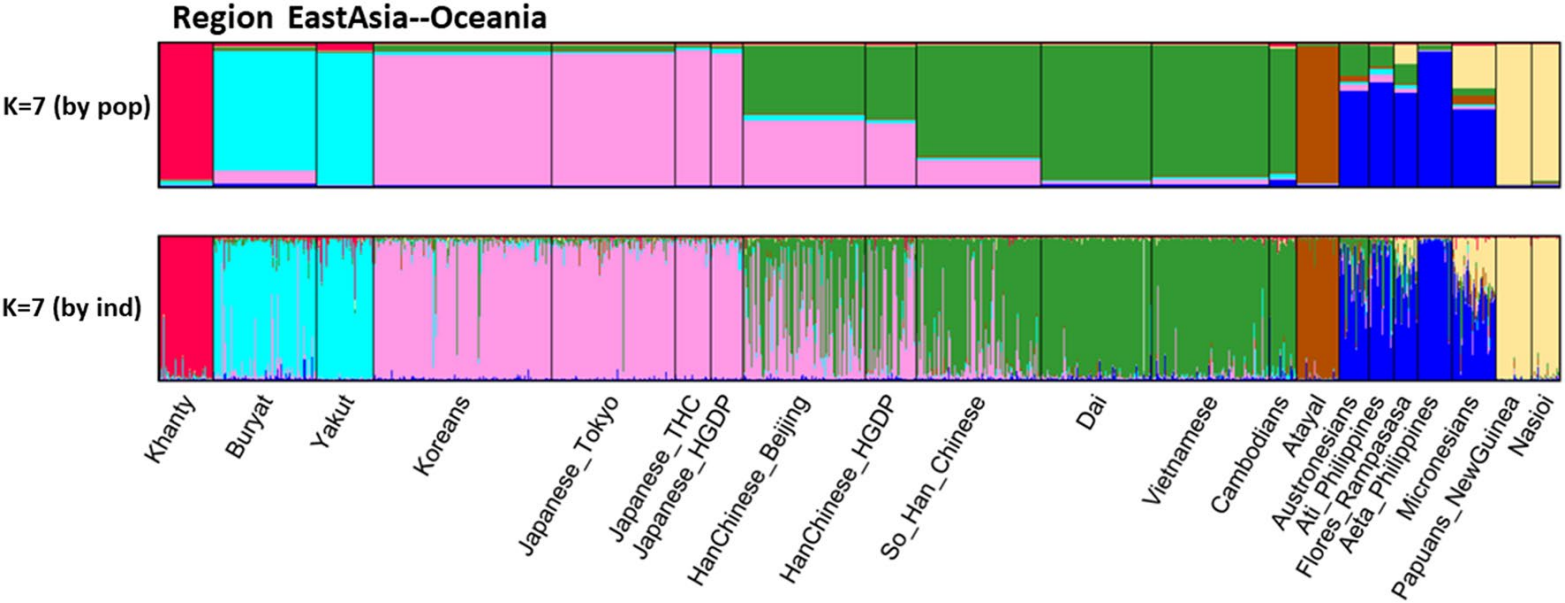
STRUCTURE of 21 Populations from East Asia to the Pacific

STRUCTURE analyses were run on all 79 population samples from *K* = 2 through *K* = 16. The first *K* value at which all major biogeographic regions are distinct is *K* = 6 (Fig. 5, Fig. S4). Those six clusters are the ones that correspond to “continental” clusters when representatives of all “continents” are present: Sub-Sahara Africa; North Africa, Southwest Asia, and Europe; South Central Asia; East Asia; the Pacific; and the Americas. These six are the commonly seen clusters from many studies based on SNPs (Soundararajan et al. 2016; Li et al. 2016; Cherni et al. 2016; Santos et al. 2016; Fondevila et al. 2017; Pakstis et al. 2017; Pakstis et al. 2019;Xavier et al. 2020), on studies of microhaplotypes (Kidd et al. 2017,2018a; Bulbul et al. 2018; Gandotra et al. 2020; Puente et al. 2020b; Staadig and Tillmar 2021), and on studies combining single SNPs and MHs (Phillips et al. 2019; Kidd et al. 2021). *K* = 6 provides a convenient basis for summarizing aspects of the data such as the MHs with the highest regional A_e_ values. *K* = 6 is also the point at which the likelihood increases with increasing K values begin to be progressively smaller until the curve is nearly flat at *K* = 14 to *K* = 16 (Fig. S5). *K* = 7 shows that these loci can begin to distinguish among the sub-Saharan Africans. Yet, when all 79 populations were analyzed up to *K* = 16, the African clustering looks identical to the *K* = 7 pattern (Fig. S6). In contrast, the East Asia pattern became much more complex at *K* = 16. This panel of 90 loci is capable of more refined STRUCTURE clustering when subsets are analyzed separately. When the 21 African and Southwest Asia populations were analyzed as a group, *K* = 6 showed five clusters within sub-Saharan Africa (Fig. 6) distinct from the Southwest Asians. When the 21 Siberian, East Asian, and Pacific populations were analyzed as a group, *K* = 7 showed the clearest set of clusters (Fig. 7).

#### PCA

**Fig. 8:**
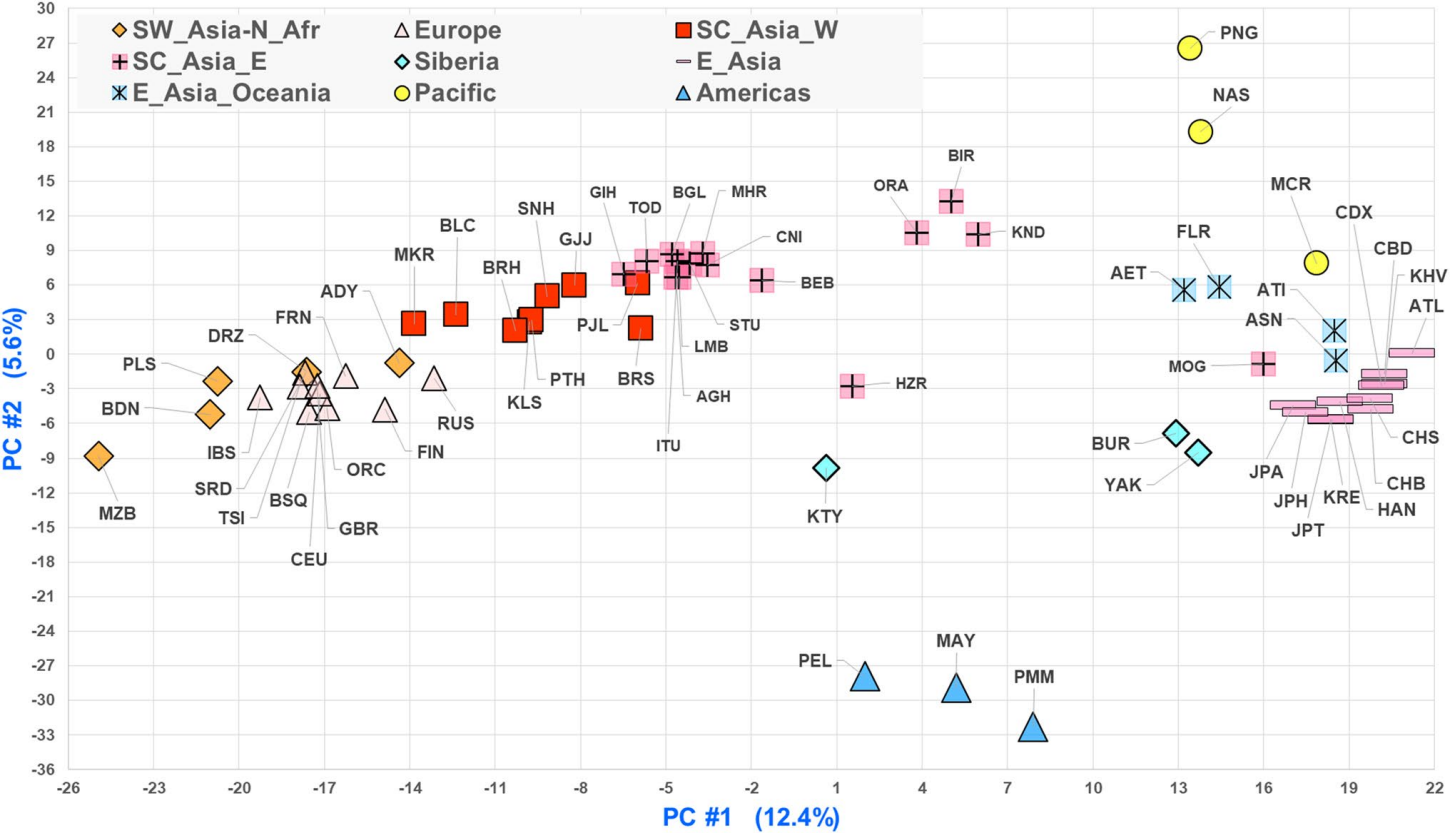
PCA of the populations after eliminating the sub-Saharan populations

The African populations are a distinct group and their distinctiveness is the primary driver of PC1 when all 79 populations are analyzed (Suppl. Fig. S7). All other populations are primarily distributed according to PC2. To separate those non-African populations better, a separate analysis was done omitting all of the sub-Saharan populations (Fig. 8). This analysis clusters the European and SW Asia populations close together at one end of PC#1 followed by the South Central Asian populations with an internal differentiation along a West-to-East axis. The Native Americans form a distinct cluster as do the East Asians. The Oceania populations form a loose cluster next to the tight East Asian cluster. The two North Asian populations (BUR and YAK) are very close together but far from the Western Siberian Khanty (KTY) which is not part of any cluster. Similarly, the Hazara (HZR) is a distinct population.

#### Tree analysis

The tree analysis of Tau genetic distances on all 79 populations involved evaluations of a total of 294 different additive tree structures of which 31 had no internal negative segments. The best of these 31 is shown in Suppl. Fig. S8. There are two small negative segments connecting the two mostly West African populations (ACB and ASW) to the African branch of the tree. This is an indication that these do not conform to the underlying assumption of an additive tree for which only random genetic drift has caused divergence of populations. Indeed, these two populations are admixed and do not meet the assumptions, but were included as part of the 1 KG set of populations.

In general, many of the clusters of populations are similar to those seen in the STRUCTURE and PCA analyses. The South Asians are divided into four different clusters in the tree. One is close to the European and SW Asia cluster; the others are more differentiated.

## Discussion

The utility/value of a locus in forensics can relate to at least four different questions: individualization, ancestry inference, kinship analysis, and mixture resolution. Individualization is often noted as the random match probability (RMP) reflecting the low likelihood that a match between evidence and an accused individual would have occurred by chance alone. Ancestry inference can be pursued as the identification of the population for which the probability of the observed genotype is highest (Kidd et al. 2018b; Rajeevan et al. 2020). The value of a panel of loci in anthropology is related to what the genetic data can tell about population relationships and histories (Kidd et al. 2021). Kinship analysis compares DNA sequence or dense markers among individuals to determine the likely degrees of relationship. Paternity testing is one form of kinship analysis. Mixture deconvolution is a developing field with probabilistic genotyping available for STR analysis but not yet for microhaplotypes. As discussed in the following sections, microhaplotypes are useful in all of these areas.

### Individualization

SNPs are overwhelmingly di-allelic and hence provide less information per locus than the polymorphic STRs when comparing a forensic sample with a reference sample. High levels of individualization measured by random match probability (RMP) are a consequence of the high A_e_ values of the loci. Figure 3 plots the RMP by population based on all 90 microhaplotypes. Although the scales are very different, Figs. 2 and 3 show otherwise similar variation among populations, because both are based on the heterozygosities of the 90 loci in the 79 populations. Both show high A_e_ values in African and significantly lower values in the Pacific and Native American populations. The range of population-specific RMP values is close to 50 orders of magnitude from a minimum of 10^–115^ to a maximum of 10^–66^. Even at the maximum value, the RMP based on all 90 loci is highly probative.

There is a significant range in the average A_e_ values (3.00–6.25) across all 79 populations among the 90 microhaplotypes (Fig. 2). While some of the loci are at the low end of the distribution overall, a relevant question is whether or not some of the better markers exist in different regions of the world. The STRUCTURE software can show reliable clusters of populations at higher *K* values (Fig. S6), but *K* = 6 provides a convenient basis for summarizing aspects of the data such as the MHs with the highest regional A_e_ values. Table S3 summarizes the top 20 MHs ranked by A_e_ value for each of the six biogeographic regions defined in Fig. 5. The averages of the average A_e_ values for the 20 highest loci are lower for the non-African regions with the smallest for the Pacific populations, but the decrease is not great compared to the overall decrease seen in Fig. 2. Overall, there are 38 different loci in this tabulation. Many of the loci have a high A_e_ in more than one broad region of the world. Only 6 of these 38 loci occur in all six biogeographic regions (cf. Figure 5) and are listed in Table 4. These are the highlighted loci in Fig. 1. The averages for those loci that rank among the top 20 are above 4.0 (See Suppl. Table S3). Many markers have good A_e_ values for random match probabilities and for mixture deconvolution for nearly all populations.

**Fig. 9:**
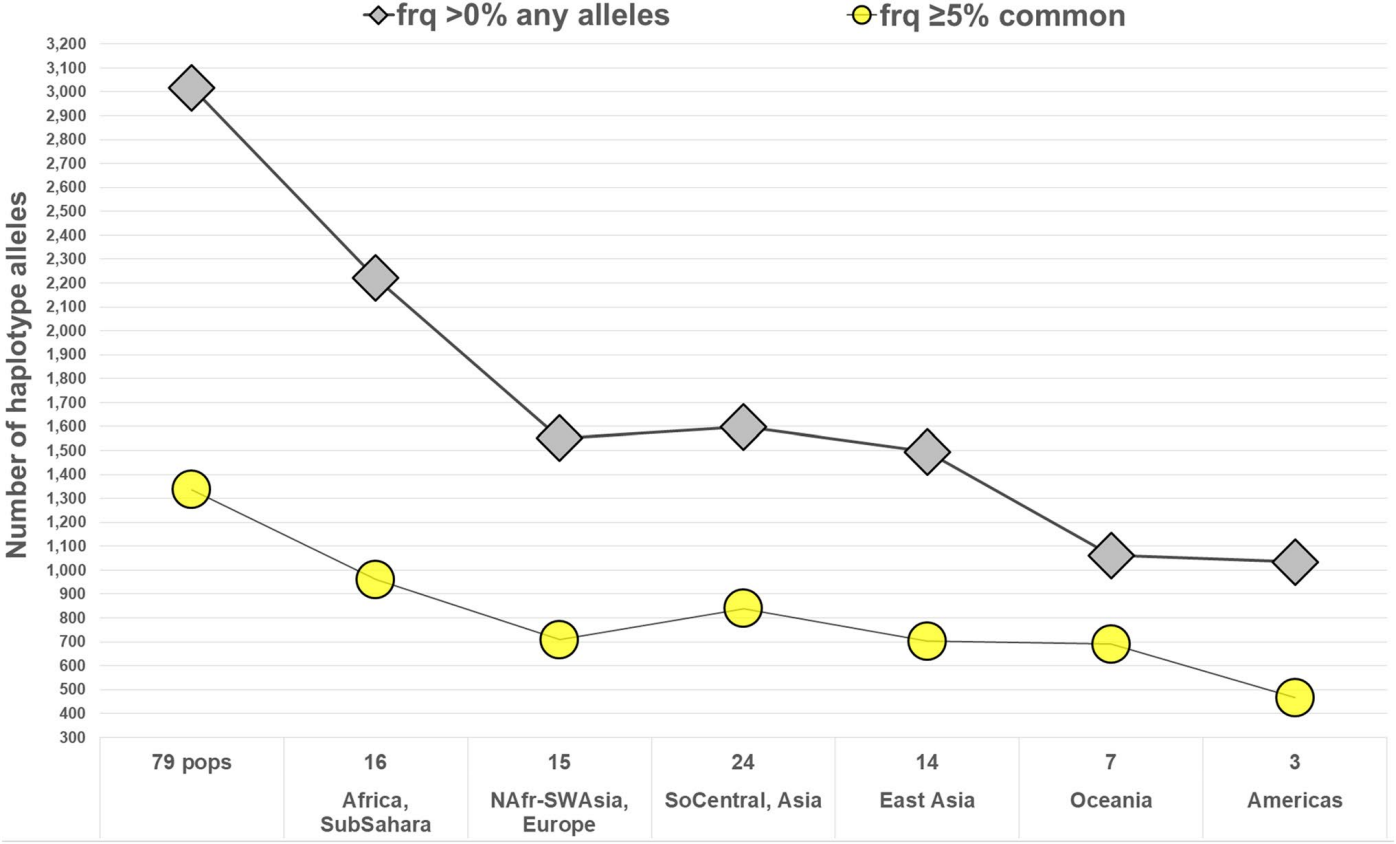
Microhaplotype alleles present and at common frequencies in specific populations for each of 6 world regions. Most of the low-frequency alleles are very rare from a global perspective

**Fig. 10:**
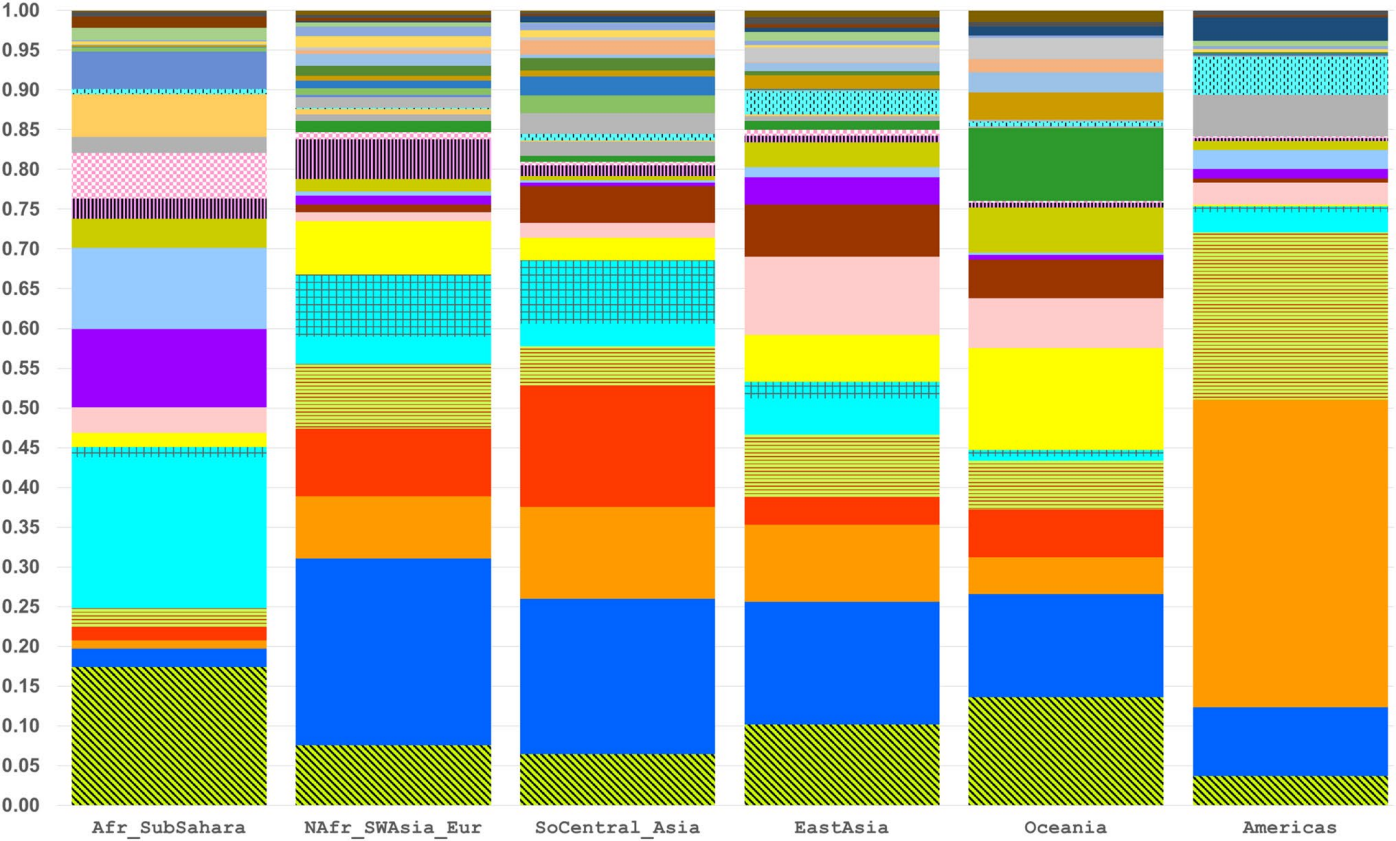
Average allele frequency bar plot for mh01KK-212 for each of 6 major biogeographic regions. This microhaplotype has the largest value for Rosenberg’s I_n_ in 79 populations (0.88; Fig. 4) and the second higheset average A_e_ for 79 populations (9.708; Suppl. Fig. S3). The 34 alleles with frequencies ≥ 5% in specific populations are plotted separately with different colors/patterns; the 58 alleles with frequencies < 5% are pooled (bars shown with black diagonal lines and green background)

**Fig. 11:**
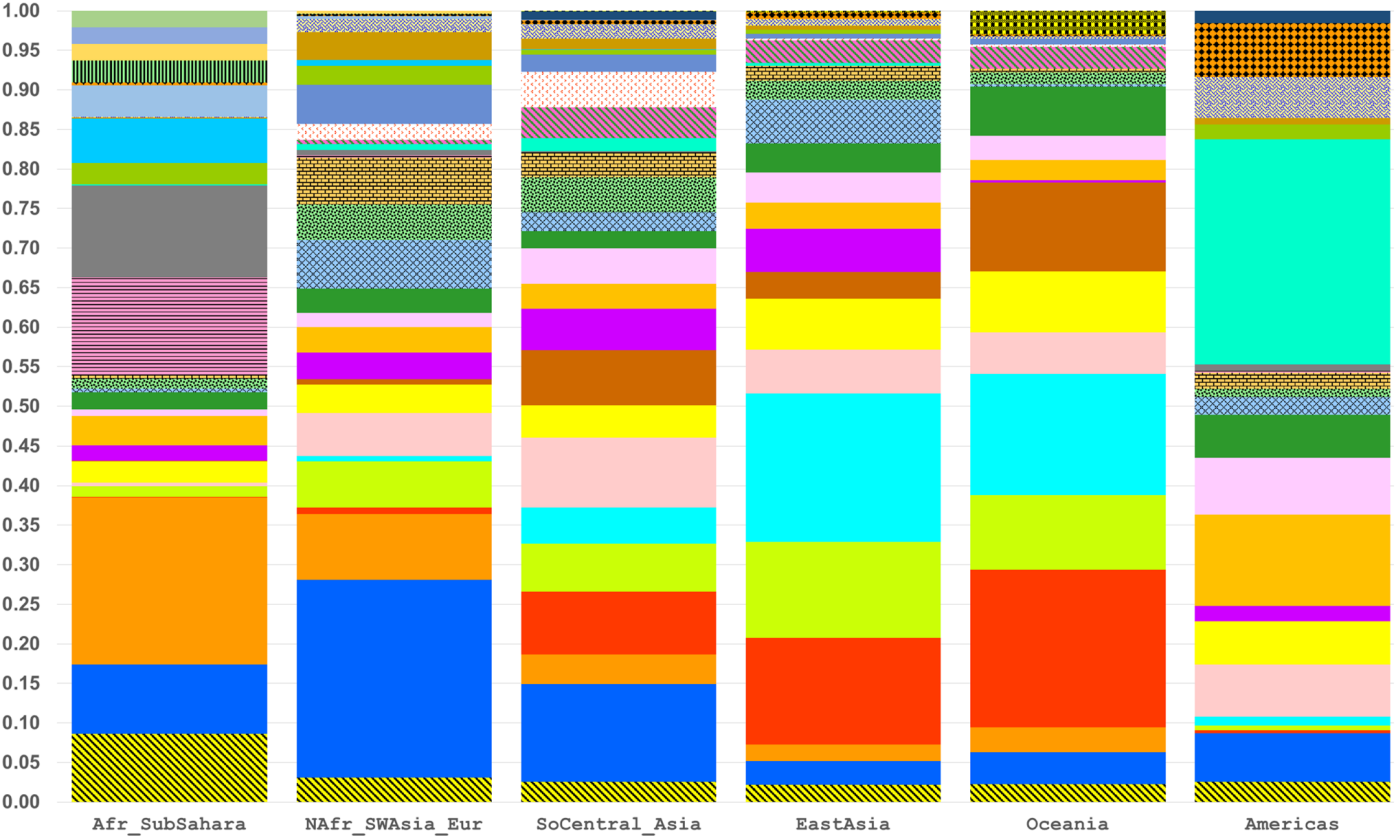
Average allele frequency bar plot for mh05KK-170 for each of 6 major biogeographic regions. This microhaplotype has the second largest value for Rosenberg’s I_n_ in 79 populations (0.81; Fig. 4) and the highest average A_e_ for 79 populations (9.750; Suppl. Fig. S3). The 33 alleles with frequencies ≥ 5% in specific populations are plotted separately with different colors/patterns; the 24 alleles with frequencies < 5% are pooled (bars shown with black diagonal lines and yellow background)

The large number of MH alleles varying in the six biogeographic regions are illustrated in Fig. 9. There are 3018 total different MH alleles in the dataset analyzed with 1337 occurring at common frequencies ≥ 5% in specific populations, while a total of 1810 MH alleles occur at frequencies > 2%. The remaining 1208 alleles mostly occur at very low (usually rare) frequencies; for example, 910 of the 1208 very-low-frequency mh-alleles are only counted to occur once or twice in the whole dataset. Supplemental Table S3 lists the 20 highest ranking MHs by average A_e_ in each of six world regions. The average MH allele frequencies in each of six major geographic regions are shown as bar plots for the microhaps, mh01KK-212 (Fig. 10) and mh05KK-170 (Fig. 11), with the highest I_n_ values (0.88 and 0.81) in 79 populations and the highest average A_e_ (9.708 and 9.750) in the 79 populations.

### Ancestry inference: population relationships

High I_n_ markers require a reference database to determine allele frequencies for calculating RMP values and for use in forensic attempts to identify the population ancestry of the donor of a DNA profile. This study provides reference data on 79 population samples. Several of those populations are smaller than ideal for forensic reference, but as seen in Fig. 5, the clusters at *K* = 6 and *K* = 7 define Mendelian populations of considerable size in some cases. It is clear that an amalgam of European population samples in one STRUCTURE cluster is as valid a reference population as a forensic reference population such as “U.S. White”.

The PCA and STRUCTURE results presented show that the extensive genetic variation in the 79 populations analyzed with the 90 MH panel can both differentiate clear population groupings for major geographical areas of the world as well as delineate distinct subgroupings of populations, especially when analyses are restricted to particular biogeographic regions.

There were no real surprises in the population relationships seen in STRUCTURE analyses and PCA. Indeed, as noted earlier, several other sets of markers on similar collections of populations have shown similar relationships (e.g., Bulbul et al. 2018) to those seen in Figs. 5 and 8. What these analyses do demonstrate is that this set of markers is highly informative for population similarities and differences at *K* values > 6. The new marker data do provide new information on some of the populations as discussed and also presented separately for African and East Asian populations below.

### Comments of overall analyses of 79 populations using these 90 microhaplotypes

The six main clusters of populations seen in Fig. 5 and Fig. S4 remained distinct at higher *K* values. Figure S5 shows that likelihoods increased through *K* = 14 but at progressively lower increases as *K* increases until likelihoods remain almost constant after *K* = 14. What happened is that the six major regions have been subdivided at the higher *K* values and the “intermediate” populations (i.e., the magnified blocks in Fig. 5) with small sample sizes have differing patterns at the higher *K* values. In supplemental material, we present analyses at *K* = 16 (Fig. S6) which is a higher *K* value than the likelihood increases warrant, but illustrates the general pattern for subdivisions of the six major regions. For Africa, the change from *K* = 7 (Fig. 5) occurred at *K* = 13 in the 79-population analysis when the Biaka Pygmies became distinct from the East Africans. That pattern persisted through *K* = 16 (Fig. S6) but with the Ethiopian Jews showing differing patterns at higher *K* levels. The North African and Southwest Asian populations became a separate cluster from the Europeans at *K* = 9 and the cluster persisted through higher *K* values. The South-Central Asia cluster separates off the Pakistani populations with a distinct admixture component at *K* = 13 and that distinction remains through *K* = 16. Three of the South-Central Asia populations show inconsistent patterns of clustering after *K* = 13. In contrast to the small refinements of the African and European patterns, the East Asian patterns became more subdivided with increasing *K* value, as discussed below. The Oceania populations show several different patterns at the different *K* values.

### Comments on African ancestry inference of these 90 microhaplotypes

Based on the overall analyses, we chose 21 populations for a more detailed analysis: the African and Southwest Asian samples. STRUCTURE analyses stabilized at *K* = 5 and *K* = 6 (Fig. 6). The Mozabites clustered with the SW Asian populations as a distinct group. The Ethiopian Jews were intermediate between the SW Asian and Sandawe from Tanzania. Other East African populations form a distinct cluster and the Central African Biaka population was distinct. The West African populations show some indication of two distinct groups with the Gambians and Mandenka distinct from both Yoruba samples and the Esan. This pattern of subdivision of the African cluster does not occur in the larger analyses of all 79 populations (Fig. S6). PCA of all 79 populations (Fig. S7) showed a distinct African cluster but no clear separation of Eastern vs. Western African populations. The Ethiopian Jews were distinct. PCA of the 21 populations showed that these populations generally are distributed along PC#1 (24.5%) as West Africa, East Africa, Ethiopian Jews, the Mozabites, and the SW Asian populations. PC#2 (9.1%) essentially separated the Biaka from all others (Suppl. Fig. S9a). PC#3 (8.2%) more clearly separated the East Africans and Ethiopian Jews from all the others (Suppl. Fig. S9b). PCA provided barely any evidence of clustering among the West African populations with only the Mandeka slightly different from the others. The two samples of admixed African-European origin cluster with the African populations by PCA but closer to the East Africans.

### Comments on East Asia and the Pacific

The most striking result for the 79 population analysis is that at *K* = 11, the three samples of Han Chinese all show an “admixture” pattern with many individuals showing mixed membership in the Northeast Asia (Koreans and Japanese) cluster and the Southeast Asia (Dai, Vietnamese, and Cambodians) cluster. That pattern persisted through *K* = 16. If it has any meaning, it is probably that the Han Chinese are intermediate in a North-to-South cline in far East Asia and not that they are individually admixed of those flanking populations. At *K* = 9, the Atayal became distinct. At *K* = 10–16, the Khanty became distinct and usually (for *K* = 10 to 14) group with the Buryat and Yakut; in both cases, they remained distinct through *K* = 16 (Fig. S6). Oceania showed inconsistent clustering among the populations except for the consistent clustering of the two Melanesian populations together.

Similar population groupings are seen in the PCA results (Fig. S10). The Khanty from northwest Siberia is a clearly distinct population in this analysis. Note that in the full global context, it was intermediate between the Europeans and East Asians. We chose 21 population samples from Western Siberia to the Pacific omitting the South Central Asian samples that were a clearly distinct cluster in Fig. 5. STRUCTURE analysis of these 21 populations showed clear clusters at *K* = 7 (Fig. 7). The Buryat and Yakut samples cluster together both in the STRUCTURE analysis of the 21 samples and in the PCA of all 79 populations (Fig. 5). The Koreans and the three samples of Japanese ancestry form a clean cluster in STRUCTURE at all K levels, but are close to the Chinese in the PCA analyses. The three Chinese samples appear admixed between the Japanese and the three South East Asia populations that form a clean cluster. The STRUCTURE data constitute evidence for a North-to-South cline of genetic differentiation in Far East Asia. The Atayal sample defined its own isolated cluster in STRUCTURE at *K* = 9, 10, and 16 but group with the South East Asian populations from *K* = 11 to 15. The various Oceania populations form a noisy cluster with evidence of admixture except for the two Melanesian samples from Papua New Guinea that are distinct at all K values in analyses of both the full (79) and restricted (21) sets of populations.

### A general comment

Overall, these 90 microhaplotype markers are a powerful set for population relationships, but it was impossible from these analyses to determine when a subset of populations would provide an answer not inferable from the full set of populations. The Africans, in the separate 21 population analysis, clearly show clustering at *K* = 5 that is not seen in any of the results for all 79 populations. In contrast, the East Asians by themselves cluster in ways that are similar (but never identical) to the clustering of all populations at *K* levels up to *K* = 16. We do not fully understand the cause in this case of the different patterns. We know that different markers are most relevant to different regions; the magnitude of the allele frequency differences is undoubtedly relevant. How well this regional inconsistency in finer clustering generalizes to other datasets is unknown at present.

### Kinship

Any multiallelic genetic system is useful for kinship analysis. Indeed, even a di-allelic locus provides evidence of relationship by allele sharing. In this respect, the high A_e_ values of this set of MHs should be especially informative, because the probability of allele sharing identical by state can be much less than sharing identical by descent for close relatives. However, no direct test has been done. Recent papers by Puente et al. (2020a), Staadig and Tillmar (2021), and Wu et al. (2021b) have assessed microhaplotypes in kinship analyses to varying degrees. Based on (Wu et al. 2021b) with 54 high I_n_ MHs that were problematic at relationships beyond second degree, we cannot expect the 90 MHs in our study to be good at distant relationships. How good the 90 will be is for future research.

### Mixture deconvolution

Three questions arise when considering the existence of mixtures in a forensic sample. First, is there a mixture? The essential proof that a mixture exists is the presence of at least three alleles at several of the loci. Note that this criterion cannot be met by a di-allelic SNP. The only way a di-allelic SNP can contribute to the inference of a mixture is if a quantitative method is used and the two alleles differ in their values, e.g., sequence read number, more than heterozygote read imbalance would explain. Second, how many contributors are there to this mixture? At any one locus, the minimum number of contributors is the number of alleles seen divided by 2 and, if a fraction, rounded to the next whole number: five alleles seen implicates 3 contributors; six alleles also implicates 3 contributors. The loci with the largest numbers of alleles seen provide an overall minimum estimate of contributors that applies to all loci. Note that sensitivity issues and diminishing concentrations with larger numbers of contributors prevents any realistic estimate of the maximum number of contributors. However, the global sum of all the alleles seen at all the loci can implicate more contributors than the maximum seen at individual loci (see Fig. 2 in Bennett et al. (2019) for an illustration). Also, quantitative variation in allele “intensity” may also provide hints at larger numbers of contributors, but some model of the relationships of numbers of copies of alleles to their intensity is required.

Finally, can the individual multi-locus genotypes of the contributors be determined? It may be possible to readily infer the contributing genotypes at a single locus using allele “intensity” (e.g., read count in MPS) as seen at locus mh05KK-170 in Bennett et al. (2019). However, the permutations of the individual locus results overwhelm such single locus approaches. This becomes an issue for probabilistic genotyping of microhaplotypes analogous to the use of STRMix (Buckleton et al. 2019) for probabilistic genotyping of forensic STR data. In the forensic case, the question is usually whether a known sample can be part of a mixture. This is a different question than attempting to fully deconvolute a mixture. This is an area that needs development for microhaplotypes because of the many variables that are involved. Elements of such deconvolution methods include the number of contributors, the relative amounts of each contributor, and the allele frequencies in the relevant population(s). The 90 MHs provide a set of highly heterozygous loci that can help with some of these issues and have the advantage of low mutation rates and the absence of stutter.

### Optimizing the panel

This panel of 90 MH loci was designed to have high A_e_ and high I_n_. This has resulted in loci with, on average, greater extent to encompass more SNPs. Eliminating the loci with the lowest A_e_ and/or I_n_ values globally should improve the efficiency of the panel. However, a careful analysis should be undertaken to assure that the lowest I_n_ marker for all populations is not providing significant differentiation of some population(s). We generated exploratory STRUCTURE runs from *K* = 2 to *K* = 8 for 79 populations after excluding 19 MH with I_n_ ≤ 0.25. The cluster patterns of the highest likelihood runs for the 71 MHs were all very similar to those obtained with all 90 MH. The most noticeable difference occurred at *K* = 7 where the Biaka from central Africa clustered with the West African groups instead of the East African cluster. Some of the excluded MH markers undoubtedly have value in differentiating among the sub-Saharan groups. Given the high level of informativeness of the panel for obtaining results at 90 loci, efficiency is not an issue. Rather, any pruning would allow space for adding additional marker loci with higher values, including some of the best of the loci identified by others, e.g., (Wu et al. 2021a), have identified many MHs with global average A_e_ values > 5.0. Those are issues for future research.

### General utility of microhaplotypes

While the loci studied here are human specific and will not be relevant to other species, the general molecular approach and methods (Gandotra et al. 2020) are applicable tools in population genetic studies of other organisms. The fields of ecology and conservation are increasingly using molecular techniques and some researchers are already using microhaplotypes (Meek and Larson 2019). Microhaplotypes have been shown to be much more informative per locus than SNPs in studying the familial relationships among Kelp Rockfish (Baetscher et al. 2018). Microhaplotypes have also been used to study porpoises (Morin et al. 2021) and salmon (Larson et al. 2016; McKinney et al. 2017). Tessema et al. (2020) identified 93 microhaplotypes in *Plasmodium falciparum*. Those *P. falciparum* microhaplotypes had a median A_e_ of 3.33 and provided good discrimination between related and unrelated polyclonal infections.

### Impact on forensic practice

In spite of their technical advantages over the forensic STR markers, SNPs have not been incorporated in routine forensic practice. Part of the reason has been the need for separate methodologies to type STR loci and SNPs. With the advent of MPS, it is now possible to use one technology and multiplex the standard STR markers with a reasonable panel of SNP-based markers in the same sequencing run. We show in this study that microhaplotypes with high A_e_, rivaling the A_e_ values for STR markers, can be found and are far superior to individual SNPs. We believe that such microhaplotypes will supplant individual SNPs in future applications. As more laboratories acquire sequencing technology, it may be possible for microhaplotypes to become a tool in forensic practice while maintaining the standard STR markers and the national databases of convicted felons. However, the costs of new equipment and training of personnel and the absence of an agreed upon panel of highly informative microhaplotypes remain major obstacles.

### Future studies

Refining and optimizing the microhaplotype markers that have already been identified for more localized geographic regions will likely be productive. Identifying additional useful microhaplotypes would be helpful. Some may emerge as more diverse human populations are studied routinely. While we have studied 79 populations from major geographical regions of the world, there is still a need to obtain better coverage of the diversity of human populations, especially in Africa, North Asia, Southeast Asia, and the Americas. Recent reviews and population genetic studies (Ramsay et al. 2021), for example, continue to indicate that the diversity of African populations is greater than what has been routinely studied. Indigenous populations of the Americas (Moreno-Estrada et al. 2014; Homburger et al. 2015; Barbieri et al. 2019) also need better coverage.

## Conclusions

Our results document this panel of microhaplotype markers as the best one so far with highest overall values of A_e_ and I_n_ in the largest number of populations studied. The combination of multiplex mMHseq) and the expanded set of populations studied from around the world revealed a highly informative set of markers that has characteristics that can serve a range of forensic, medical, and anthropological applications. Additional useful microhaplotypes will likely emerge from other and future studies (e.g., Wu et al. 2021a). New analyses can focus on tailoring the best subsets and supersets of MH markers for use in specific geographical regions as well as for major world regions. As more extensive sampling and analyses of world populations occur, it can be expected that the ability to distinguish more refined population relationships in multiple world regions will increase, especially in Africa.

## Supplementary Information

Below is the link to the electronic supplementary material.

Supplementary file1 (pdf 2762 KB)

## Data Availability

Genotype profiles on 90 MHs for 556 individuals in 16 Kidd lab population samples (including the 524 sequenced Kidd lab individuals and 32 individuals from HGDP studies of the same population samples) have been deposited in the Zenodo archive and can be freely accessed at https://doi.org/10.5281/zenodo.5095364. Data for the additional individuals included in the analyses were taken from public datasets as indicated in the text. The mMHseq 90-plex data for 524 sequenced individuals from 16 Kidd lab population samples are available at the Scharfe lab website, https://mmhseq.shinyapps.io/mMHseq.
