# Supplementary material for: The population genetics characteristics of a 90 locus panel of microhaplotypes
Source: Hum Genet. 2021 Oct 13;140(12):1753–73. doi: 10.1007/s00439-021-02382-0 (PMC8553733; doi:10.1007/s00439-021-02382-0)

Supplementary Material

for manuscript

The population genetics characteristics of a 90-locus panel of microhaplotypes

Andrew J. Pakstis[1], Neeru Gandotra[1], William C. Speed[1], Michael Murtha[1], Curt Scharfe[1], Kenneth K. Kidd[1]

[1] Department of Genetics, Yale University School of Medicine, New Haven, CT 06520

Index of Table and Figure Legends

Table S1. SNPs for each microhaplotype, chromosome locations, and alleles.

Table S2. Quality control (QC) metrics.

Table S3. The top ranked 20 loci by average A_e in each of 6 world regions. Blue highlighting identifies the 6 best microhaplotypes in Table 4 to show how they rank in the 6 separate regions. There are 38 different microhaplotypes present in this table.

Figure S1. Quality control of 384 samples sequenced using mMHSeq assay**.**

Figure S2. Distribution of average reads per genotype per individual for the 90 microhaplotype loci**.**

Figure S3. Average Ae in 79 populations for each of 90 microhaps.

Figure S4. STRUCTURE individual bar plot at K=6 & 7 for all 79 populations

Figure S5. Likelihoods for STRUCTURE runs K=2 to 16 for 90 mh, 79 population dataset.

Figure S6. Individual bar plot of highest likelihood run at K=16 for the STRUCTURE analysis of 79 populations in the 90 microhaplotype dataset.

Figure S7. PCA of all 79 populations

Figure S8 The best least squares (LS) population tree. A total of 294 different trees were evaluated.

Figure S9a. PCA (PC1 x PC2) of the Africa – Southwest Asia subset of 21 populations

Figure S9b. PCA (PC1 x PC3) of the Africa – Southwest Asia subset of 21 populations

Figure S10. PCA of the Siberia – East Asia - Pacific subset of 21 populations

Table S1. SNPs for each microhaplotype, chromosome locations, and alleles.

	Microhap	Chr	Molecular Extent (basepairs)	# SNPs	Nucleotide Position (GRCh37)		dbSNP rs-number	Alleles	
1	mh01KK-172	1	226	8	1486834		rs3128342	C	A
					1486880		rs115309688	C	T
					1486903		rs3766176	T	C
					1486924		rs538618101	T	C
					1486939		rs3766175	A	T
					1486946		rs531720810	G	A,C
					1487043		rs74511912	C	T
					1487059		rs1887284	G	A,T
2	mh01KK-001	1	283	11	3743109		rs2275834	A	G
					3743132		rs4648344	T	C
					3743150		rs574831779	C	T
					3743151		rs4648345	G	A
					3743186		rs114208229	C	T
					3743223		rs376407444	C	T
					3743319		rs6663840	G	A
					3743337		rs199565833	G	A
					3743350		rs58111155	G	C
					3743367		.	C	A
					3743391		rs6688969	C	T
3	mh01KK-205	1	242	9	18722692		rs11810587	T	C
					18722713		rs1336130	T	C
					18722730		rs1336822916	C	T
					18722737		rs1336131	G	T
					18722801		rs1533623	G	A
					18722846		rs1533622	G	A
					18722870		rs547666214	C	T
					18722871		rs138362286	G	A
					18722933		rs56176731	G	C
4	mh01KK-212	1	243	17	202616547		rs16850184	G	C,A
					202616580		rs186753019	G	A
					202616589		rs57658975	C	A
					202616590		rs72750624	C	G,A,T
					202616591		.	C	A,T,G

	Microhap	Chr	Molecular Extent (basepairs)	# SNPs	Nucleotide Position (GRCh37)		dbSNP rs-number	Alleles	
					202616596		rs909997628	C	T
					202616597		rs114711668	G	A
					202616616		rs11589785	A	G
					202616637		rs58023111	T	C
					202616655		rs892141277	T	C
					202616656		rs369518303	G	A
					202616676		rs59815839	T	C
					202616687		rs58060874	T	C
					202616697		rs569670376	C	A
					202616698		rs114630930	C	T
					202616765		.	G	A
					202616789		rs12121078	C	T
5	mh01KK-117	1	189	9	204633340		rs17413714	A	C
					204633351		rs923492268	C	T
					204633397		rs2772234	A	G
					204633443		rs551532732	G	A
					204633462		.	T	C
					204633500		rs1610401	G	C
					204633502		rs949532333	C	T
					204633526		rs1610400	C	T
					204633528		rs556261613	G	T
6	mh01NK-001	1	280	5	230820351		rs2479135	A	G
					230820446		rs1402249007	T	C
					230820578		rs2296796	G	A
					230820605		rs2296797	G	T
					230820630		rs2296798	G	A
7	mh01KK-213	1	216	16	232811740		rs79793450	T	A
					232811769		rs7521883	G	C
					232811780		rs6424246	T	C
					232811797		rs75030648	G	A
					232811814		rs1402593093	G	C
					232811821		rs9424470	G	C
					232811830		.	A	T
					232811831		.	A	G
					232811834		.	T	A

	Microhap	Chr	Molecular Extent (basepairs)	# SNPs	Nucleotide Position (GRCh37)		dbSNP rs-number	Alleles	
					232811837		.	A	T
					232811839		.	T	G
					232811847		rs186507293	G	A
					232811909		rs12118941	C	A
					232811927		rs993762397	C	T
					232811928		rs77211663	T	C
					232811955		rs12120379	A	G
8	mh02KK-022	2	249	9	3172438		rs13021132	C	T
					3172539		rs79989676	A	G
					3172582		rs111869331	G	A
					3172597		rs74618846	G	A
					3172635		rs10495516	G	A
					3172653		rs10495515	G	A
					3172658		.	C	T
					3172682		rs751294754	G	A
					3172686		rs62123466	A	G
9	mh02KK-138	2	249	8	46191983		rs4953292	G	A
					46191988		.	A	G
					46192017		rs2595202	T	G
					46192078		rs6759301	T	C
					46192092		rs59298278	A	G
					46192182		rs373632365	A	G
					46192191		rs2595203	G	A
					46192231		rs6715568	A	G
10	mh02KK-029	2	236	14	69138957		rs1036977184	T	C
					69138958		rs77788766	G	A
					69138982		rs191237929	C	T
					69138983		rs1056213077	C	T
					69138986		rs372620871	C	T,A,I
					69138992		rs6726044	C	A
					69139026		rs10191936	C	A
					69139137		rs12999343	A	G
					69139150		rs115054914	C	T
					69139155		rs78044414	A	G
					69139157		rs72905669	T	C
					69139167		rs114774962	A	G

	Microhap	Chr	Molecular Extent (basepairs)	# SNPs	Nucleotide Position (GRCh37)		dbSNP rs-number	Alleles	
					69139185		rs573151267	G	A
					69139192		rs138590193	A	G
11	mh02KK-013	2	221	9	105833031		rs114422847	C	T
					105833083		rs147490454	C	G
					105833093		rs7584136	A	C
					105833116		rs945032143	A	T
					105833128		rs35414072	A	G
					105833193		rs34544149	C	T
					105833235		rs1978821	C	T
					105833236		rs367849909	G	A
					105833251		rs1978820	T	C
12	mh02KK-031	2	252	14	123395790		.	T	C
					123395799		rs199643559	G	A
					123395805		rs182716348	G	A
					123395825		rs6740521	T	C
					123395831		.	T	C
					123395877		rs13400652	C	T
					123395915		.	T	C
					123395921		rs13400673	C	T
					123395943		.	A	G
					123395956		rs185967717	C	T
					123395957		.	G	A,T
					123395981		rs1056613831	G	A
					123396005		rs7575942	C	T
					123396041		.	G	A
13	mh02KK-134	2	104	8	161079411		rs12469721	A	T
					161079435		rs3101043	T	C
					161079450		rs3111398	C	T
					161079456		rs190062220	C	T
					161079463		rs535658612	A	G
					161079478		.	C	T
					161079498		rs555403336	C	T
					161079514		rs72623112	G	A
14	mh02KK-136	2	198	7	228092334		rs6728149	A	G

	Microhap	Chr	Molecular Extent (basepairs)	# SNPs	Nucleotide Position (GRCh37)		dbSNP rs-number	Alleles	
					228092338		.	C	T
					228092351		rs184900040	C	T
					228092389		rs6714835	T	G
					228092406		rs6756898	C	T
					228092459		rs12617010	C	A
					228092531		rs905305021	T	A
15	mh02KK-014	2	239	16	228524072		rs73084811	G	A
					228524087		rs4270334	T	C
					228524094		rs78561058	T	C
					228524110		rs6730730	A	G
					228524141		rs72961055	G	C,T
					228524154		rs60403543	C	T
					228524162		.	C	G
					228524165		rs58129342	G	A
					228524166		rs182570717	T	G
					228524169		.	C	T
					228524177		rs77155305	A	G
					228524179		rs4332915	G	A
					228524222		rs997910796	G	A
					228524254		rs4580373	A	G
					228524309		rs139811713	G	A
					228524310		rs4321359	T	C
16	mh02KK-015	2	221	11	240004773		rs75104001	C	T
					240004807		rs3791372	T	C
					240004838		rs3791373	A	G
					240004849		rs868173690	A	G
					240004891		rs3791374	A	G
					240004900		rs3791375	G	A
					240004922		rs74566136	C	T
					240004923		rs1385754794	G	A
					240004924		rs112145050	C	T
					240004961		rs28404128	A	G
					240004993		rs140587940	A	C
17	mh03KK-016	3	201	12	14377432		rs367833150	C	T
					14377443		rs9310444	T	G
					14377469		rs9310445	C	T

	Microhap	Chr	Molecular Extent (basepairs)	# SNPs	Nucleotide Position (GRCh37)		dbSNP rs-number	Alleles	
					14377500		rs9867515	C	T
					14377505		rs9867516	C	T
					14377510		rs375235722	T	G
					14377518		rs968586706	A	G
					14377544		.	C	A
					14377589		rs13082821	G	T
					14377609		rs874969	C	T
					14377631		rs75565440	C	T
					14377632		rs874967	G	T
18	mh03KK-017	3	179	7	37516028		rs140794158	C	T
					37516042		rs576367436	C	T
					37516078		rs150108526	G	A
					37516083		rs267527	A	G
					37516136		rs267526	A	G
					37516184		rs2162683	C	T
					37516206		rs197766	G	A
19	mh03KK-047	3	243	7	45166218		rs542886330	C	T
					45166223		rs6441891	T	C
					45166253		rs59145932	C	T
					45166285		rs7612333	G	A
					45166288		rs1044919501	G	A
					45166416		rs34703630	C	T
					45166460		rs189841224	G	A
20	mh03KK-018	3	224	13	117156240		rs6806321	G	A
					117156255		rs9870146	C	T
					117156290		rs1576298529	C	T
					117156293		rs77141935	T	G
					117156294		rs3971402	C	A
					117156327		rs116231064	G	A
					117156336		rs1341538891	G	A
					117156355		rs374027602	G	T
					117156384		rs9832546	A	G
					117156402		rs6770710	T	C
					117156422		rs568503846	C	T
					117156432		rs3971401	A	C
					117156463		rs12695349	T	C

	Microhap	Chr	Molecular Extent (basepairs)	# SNPs	Nucleotide Position (GRCh37)		dbSNP rs-number	Alleles	
21	mh03KK-150	3	185	9	131645972		rs1225051	G	A
					131645993		.	T	C
					131646001		rs1225050	G	A
					131646010		.	A	T
					131646024		rs914956212	G	T
					131646043		rs183934495	G	A,T
					131646087		rs1225049	C	T
					131646149		rs553856529	C	T
					131646156		rs1225048	C	A
22	mh04KK-010	4	261	8	1986720		rs554349557	G	T
					1986750		rs376745306	C	G
					1986783		rs577194830	C	T
					1986824		rs545888757	G	A
					1986853		rs185470596	C	T
					1986938		rs3135123	G	A
					1986972		rs495367	A	G
					1986980		rs547950691	C	T
23	mh04KK-030	4	284	9	3666211		rs148696985	G	A
					3666283		rs539610102	C	T
					3666286		rs1227103666	T	A
					3666370		rs16844737	T	C
					3666393		rs1040225118	C	T
					3666403		rs4916615	C	T
					3666471		rs1884412	A	G
					3666484		rs1884411	C	G
					3666494		rs58827274	C	G
24	mh04KK-013	4	201	8	68444102		rs13131164	C	A
					68444111		rs944702357	G	A
					68444175		rs1262735809	C	G
					68444180		rs3775866	G	A
					68444192		rs11725922	G	A
					68444231		rs73829227	T	C
					68444257		rs3775867	G	A
					68444302		rs17088476	T	C

	Microhap	Chr	Molecular Extent (basepairs)	# SNPs	Nucleotide Position (GRCh37)		dbSNP rs-number	Alleles	
25	mh05KK-169	5	234	7	1898501		rs260410	T	A
					1898598		rs62335822	C	A
					1898603		rs62335823	G	A
					1898610		rs116092316	C	A
					1898614		rs10428542	C	T
					1898679		rs10428597	A	C
					1898734		rs183435881	T	A
26	mh05KK-170	5	256	14	2447910		rs116278333	C	T
					2447924		rs143851316	G	A
					2447928		rs436910	A	G
					2447936		rs199671779	G	A
					2447938		rs79374881	G	A
					2447946		rs58296930	G	A
					2447984		rs539631028	A	G
					2447990		rs376025308	A	G
					2448006		rs80244780	T	C
					2448024		rs74865590	C	T
					2448052		rs438055	A	G
					2448146		rs370672	G	A
					2448160		rs6555108	A	G
					2448165		rs143205094	T	G
27	mh05KK-020	5	199	7	38881438		rs80052994	C	T
					38881460		rs617938	G	T
					38881538		rs2278325	C	T
					38881599		rs180895008	T	C
					38881603		rs2278324	G	T,A
					38881628		rs525735	G	T
					38881636		rs74924445	A	C
28	mh05KK-178	5	231	9	67309764		rs35138278	A	G
					67309775		rs282290	T	C
					67309802		rs115575506	T	C
					67309818		rs75963146	A	G
					67309820		rs868867723	C	G
					67309895		rs754074051	G	A

	Microhap	Chr	Molecular Extent (basepairs)	# SNPs	Nucleotide Position (GRCh37)		dbSNP rs-number	Alleles	
					67309926		rs282291	C	A
					67309933		rs1437125	G	C
					67309994		rs116644437	A	G
29	mh06KK-090	6	280	17	29937692		rs113213828	A	G
					29937721		rs9260770	G	C
					29937740		rs2256539	C	T
					29937784		rs2523972	T	C
					29937790		rs6911940	A	G
					29937794		rs4713276	C	G
					29937795		rs1061537	G	A
					29937826		rs1061536	T	C
					29937833		rs2256543	T	C
					29937838		.	C	T
					29937845		.	C	T
					29937870		rs1250847663	T	C
					29937896		rs3202637	C	T
					29937918		rs201669651	A	C
					29937924		rs1061535	T	C
					29937949		rs1582084460	G	A
					29937971		rs6939037	T	C
30	mh06KK-104	6	188	5	165798851		rs16897782	T	C
					165798894		rs220807	C	T
					165798940		rs375807743	G	C
					165798963		rs9457084	A	G
					165799038		rs220806	C	T
31	mh06KK-008	6	275	14	169656029		rs6930377	T	G
					169656044		rs1018177853	C	T
					169656079		rs6921774	G	C
					169656120		rs6605524	A	G
					169656126		rs1488014151	C	T
					169656141		.	C	G
					169656144		rs6422749	A	G
					169656173		rs115242802	T	G
					169656216		rs571288861	G	A
					169656248		rs6605523	A	G
					169656271		rs55995725	G	A

	Microhap	Chr	Molecular Extent (basepairs)	# SNPs	Nucleotide Position (GRCh37)		dbSNP rs-number	Alleles	
					169656291		rs1016655002	G	A
					169656293		rs147272602	G	T
					169656303		rs113888184	G	C
32	mh07KK-009	7	182	16	18861121		rs13244868	C	G,A
					18861122		rs141872881	G	T
					18861136		rs958860091	G	A
					18861138		rs28401174	T	C,I
					18861140		rs2520361	A	G,I
					18861147		rs2588633	A	G,I
					18861169		rs115971745	C	T
					18861172		rs189222341	G	T
					18861174		rs528829633	A	G
					18861181		rs28465160	G	A
					18861190		.	T	G
					18861199		rs148036593	A	G
					18861219		rs28636374	A	G
					18861238		rs375413102	T	G
					18861265		rs28712504	T	C
					18861302		.	A	C
33	mh08KK-039	8	228	18	3516789		rs1215564347	C	T
					3516792		rs1905100	C	T
					3516794		rs553064422	A	G
					3516797		rs74752104	T	C
					3516836		rs10503214	T	C
					3516842		rs372991914	C	A
					3516853		rs537222635	A	T
					3516860		rs144221602	T	A
					3516871		rs184503746	C	A
					3516874		rs7822812	A	G
					3516888		rs7839517	C	G
					3516901		rs60536879	T	G
					3516908		rs531059344	T	G
					3516923		rs922795	G	A,T
					3516984		rs922794	C	T
					3516996		rs140037624	G	T
					3517004		rs7838695	G	C
					3517016		.	C	T

	Microhap	Chr	Molecular Extent (basepairs)	# SNPs	Nucleotide Position (GRCh37)		dbSNP rs-number	Alleles	
34	mh08KK-131	8	227	15	5461399		.	C	A
					5461409		rs11782143	C	A
					5461414		rs190035804	G	A
					5461418		rs140864246	G	A
					5461431		.	T	C
					5461482		rs11775090	G	A
					5461507		rs11136908	A	G
					5461517		rs67207258	C	T
					5461520		rs150466498	G	A
					5461542		rs11136909	C	T
					5461562		rs72624088	G	C
					5461574		rs117526954	C	T
					5461616		.	T	G
					5461619		.	C	G
					5461625		.	G	C
35	mh08KK-137	8	195	12	31083232		rs35052638	C	A,T
					31083256		rs1042040465	G	A,T
					31083266		rs73232805	A	G
					31083308		rs58875218	T	C
					31083309		rs539275508	C	A
					31083315		rs540725533	C	T
					31083320		rs1027527192	A	G
					31083330		rs59644946	T	C
					31083337		rs62506140	T	C
					31083381		rs113565897	C	T
					31083414		rs62506141	C	T,I
					31083426		rs184519930	A	G
36	mh09KK-161	9	289	10	344087		rs7045710	T	G
					344096		rs57160652	C	G
					344100		rs4741822	C	G
					344127		rs76724192	G	A,D
					344229		rs4741823	C	T
					344238		rs1405718576	G	A
					344286		rs192812786	G	T
					344326		rs184018458	C	G
					344332		rs16932430	T	G
					344375		rs75177501	G	T

	Microhap	Chr	Molecular Extent (basepairs)	# SNPs	Nucleotide Position (GRCh37)		dbSNP rs-number	Alleles	
37	mh09KK-010	9	264	10	2288476		rs114987572	G	A
					2288559		rs1253755319	C	T
					2288588		rs1408328	C	T
					2288633		rs1535837	G	A,T
					2288647		rs1408329	G	A
					2288677		rs11789647	T	C
					2288700		rs12555748	C	T
					2288710		rs1535838	G	A
					2288718		rs1408330	C	A
					2288739		rs189134433	C	T
38	mh09KK-145	9	218	9	4763309		rs59428916	G	A
					4763342		rs73401750	C	G
					4763343		rs181550768	G	A
					4763368		rs409950	C	A
					4763408		.	G	T
					4763473		rs12003360	A	G
					4763484		rs10815071	G	A
					4763491		rs12005199	G	A
					4763526		rs7849565	G	C
39	mh09KK-153	9	247	7	103969642		rs62576887	C	T
					103969679		rs1293747219	C	G
					103969712		rs775269370	C	T
					103969740		rs10125791	T	C
					103969775		rs2987741	A	G
					103969852		rs7047561	A	C
					103969888		rs1156731	C	G,T
40	mh09KK-157	9	155	7	135862478		rs370406708	C	T
					135862479		rs606141	G	A
					135862495		rs8193001	C	T
					135862508		rs536230806	C	T
					135862563		rs56256724	C	T
					135862592		rs2073578	A	C
					135862632		rs633153	C	T

	Microhap	Chr	Molecular Extent (basepairs)	# SNPs	Nucleotide Position (GRCh37)		dbSNP rs-number	Alleles	
41	mh10KK-162	10	266	13	3160652		rs79563339	C	G,D
					3160661		rs890873901	A	G
					3160726		rs9423462	T	C
					3160772		rs12777199	T	C
					3160775		rs9423463	A	G
					3160778		rs548253572	C	A
					3160828		rs549784993	C	G
					3160847		rs4881098	G	A
					3160856		rs1194089900	C	T
					3160857		rs112639679	G	A
					3160904		rs3829911	C	T
					3160913		rs3829912	G	A
					3160917		rs3829913	T	C
42	mh10KK-167	10	222	8	12545332		rs45594139	C	T
					12545370		rs1111062	T	C
					12545384		rs1111063	T	C
					12545407		rs370497100	C	T
					12545471		rs1010306779	G	A
					12545501		rs2815663	G	C
					12545544		rs2815662	A	G
					12545553		rs146279227	G	A,T
43	mh10KK-170	10	190	7	78910042		rs2250841	G	T
					78910043		.	C	G
					78910074		rs2250840	A	G
					78910075		.	T	G
					78910080		rs796118208	G	A
					78910101		rs990544028	T	C
					78910231		rs12359688	A	G
44	mh11KK-180	11	271	12	1690714		rs377150512	A	G
					1690725		rs7109697	T	G
					1690770		rs113739994	G	A
					1690791		rs12802112	A	G
					1690825		rs7105950	A	C
					1690852		rs996856952	C	A
					1690887		rs12360952	T	C

	Microhap	Chr	Molecular Extent (basepairs)	# SNPs	Nucleotide Position (GRCh37)		dbSNP rs-number	Alleles	
					1690911		rs28631755	A	C
					1690950		rs4752778	C	T
					1690962		rs74047734	G	A
					1690969		rs7112918	T	C
					1690984		rs4752777	C	G
45	mh11KK-181	11	128	10	2819168		rs935487257	C	T
					2819172		rs988238975	G	A
					2819175		rs718546312	A	T
					2819193		rs372212221	C	T
					2819194		rs3852525	G	A
					2819208		rs1037777782	C	T
					2819227		rs233440	G	C
					2819259		rs78013	T	G
					2819282		rs163173	T	A
					2819295		rs116463395	G	T
46	mh11KK-183	11	217	12	20020042		rs149151952	T	C
					20020094		rs56114506	A	G
					20020097		rs4757879	G	T
					20020144		rs143260920	C	T
					20020166		rs2403552	C	T
					20020176		.	C	T
					20020187		rs139023539	G	C,A
					20020192		rs573725720	G	A
					20020228		rs112906811	T	A
					20020251		rs11025352	C	G,D
					20020256		.	C	G,T
					20020258		rs2896591	T	G,D
47	mh11KK-190	11	224	7	97176319		rs1426651	T	C
					97176369		rs56058681	C	T
					97176449		rs114239369	A	T
					97176479		rs7936934	T	C
					97176482		rs61894297	G	T
					97176507		rs72968980	A	G
					97176542		rs149859006	T	C

	Microhap	Chr	Molecular Extent (basepairs)	# SNPs	Nucleotide Position (GRCh37)		dbSNP rs-number	Alleles	
48	mh11KK-191	11	190	7	99880163		rs12421109	T	C
					99880197		.	G	A
					99880224		rs12289401	A	G
					99880282		rs12420819	A	G
					99880334		rs1315919758	G	A
					99880341		rs11222337	A	C,G
					99880352		rs770566	T	C
49	mh12KK-199	12	209	8	12229744		rs1264593829	G	A
					12229786		rs17819677	C	G
					12229849		rs1641719	C	T
					12229850		rs1641720	T	G,A
					12229886		rs139574449	C	T
					12229908		rs982062076	G	T
					12229914		rs150012654	C	T
					12229952		rs6488494	C	T
50	mh12KK-201	12	177	15	27800327		rs11049080	C	T
					27800334		rs11049081	T	C
					27800348		rs7972446	G	C
					27800353		rs11834107	C	T
					27800374		rs7138224	T	C
					27800382		rs140345281	G	C
					27800386		rs7959051	T	C,I
					27800402		rs73084353	G	A
					27800404		rs73084354	A	G
					27800421		rs145035089	A	G
					27800431		rs566851642	C	A
					27800435		rs534240196	A	G
					27800446		rs7959164	T	C
					27800466		rs535383668	C	A
					27800503		rs7959186	T	C
51	mh12KK-202	12	154	5	30170229		rs10506052	A	C
					30170306		rs4931233	G	A
					30170334		rs116560588	A	T
					30170359		rs10506053	T	C
					30170382		rs4931234	T	C

	Microhap	Chr	Molecular Extent (basepairs)	# SNPs	Nucleotide Position (GRCh37)		dbSNP rs-number	Alleles	
52	mh12KK-046	12	289	8	118889488		rs1503767	T	G,A
					118889525		rs867312187	T	G
					118889528		rs75554846	T	C
					118889559		rs11068953	G	A
					118889563		rs368447602	C	A
					118889568		rs139873540	G	T
					118889681		rs557407136	C	T
					118889776		rs3903095	C	T
53	mh12KK-209	12	191	7	130308483		rs764902	A	G
					130308491		rs113507589	A	C
					130308528		rs147809360	G	A
					130308529		rs79004033	C	T
					130308630		rs5012151	G	T
					130308640		rs5012152	G	A
					130308673		rs2173910	G	A
54	mh13KK-213	13	273	11	23765409		rs186130261	A	G
					23765421		rs574786958	C	T
					23765535		rs991203428	A	G
					23765541		rs8181845	C	T
					23765546		rs8181836	G	A
					23765549		rs77728659	A	G
					23765562		.	A	T
					23765598		rs2152726	A	G
					23765601		rs2152727	T	C
					23765635		rs679482	C	A
					23765681		rs9510616	G	A
55	mh13KK-215	13	242	10	36451857		rs369450802	G	A
					36451881		rs1539549	C	T
					36451919		.	G	T
					36451930		rs4146589	T	C
					36451931		rs750651238	G	A
					36451936		rs1539548	T	A
					36452014		rs562736736	A	G
					36452056		.	C	A

	Microhap	Chr	Molecular Extent (basepairs)	# SNPs	Nucleotide Position (GRCh37)		dbSNP rs-number	Alleles	
					36452069		rs1750921	A	G
					36452098		rs1539547	C	G
56	mh13KK-217	13	235	10	46865888		rs76839632	A	G
					46865905		rs1439024173	T	C
					46865930		rs7320507	A	G
					46865944		rs1294979376	C	T
					46865970		rs9562648	G	A
					46866020		rs2764588	T	G
					46866059		rs9534373	G	A
					46866084		rs9562649	C	T
					46866096		rs60234592	A	G
					46866122		rs2765614	A	G
57	mh13KK-218	13	263	7	54060710		rs1450180563	C	T
					54060720		rs9536428	C	G,A
					54060820		rs565064657	G	A
					54060827		rs1927847	T	C
					54060881		rs9536429	T	C
					54060892		rs7492234	T	C
					54060972		rs9536430	C	T
58	mh13KK-225	13	207	7	66712622		rs1033357381	G	A
					66712629		rs539501789	C	G
					66712732		rs4884651	G	A
					66712751		rs115260715	A	T
					66712790		rs9529023	A	C
					66712815		rs4884652	C	T
					66712828		rs7329287	G	A
59	mh13KK-221	13	253	12	101759088		rs61973993	T	C
					101759111		rs1018721213	G	T
					101759119		rs602728	T	C
					101759143		rs113732191	A	G
					101759144		rs9557588	T	A
					101759165		rs7993431	T	C
					101759198		rs112552144	A	G
					101759205		rs114169269	T	G

	Microhap	Chr	Molecular Extent (basepairs)	# SNPs	Nucleotide Position (GRCh37)		dbSNP rs-number	Alleles	
					101759217		rs608555	C	A
					101759287		rs11843065	A	T
					101759330		rs114345885	A	G
					101759340		rs9300648	T	C
60	mh13KK-222	13	252	13	106642644		.	A	G
					106642650		.	T	C
					106642687		rs2248394	C	T
					106642725		rs563358657	G	A
					106642741		rs2248391	A	G
					106642744		rs180754893	T	C
					106642766		rs9583061	T	A
					106642794		rs61972611	C	T
					106642810		rs2248388	T	C
					106642815		rs79323262	T	G
					106642880		rs2391263	C	A
					106642885		.	T	G
					106642895		rs2476747	G	C
61	mh13KK-223	13	237	11	110806689		rs1192203	T	C
					110806699		rs1192204	T	C
					110806711		.	G	T
					110806742		rs1192205	C	G
					110806759		rs3825483	C	T
					110806783		rs373101786	G	A
					110806786		rs115278730	G	A
					110806789		rs3825482	G	T
					110806833		.	G	A
					110806852		rs3825481	T	C
					110806925		rs377669422	G	A
62	mh14KK-227	14	215	10	52334089		rs1953862	A	G
					52334164		rs57928440	T	C
					52334203		rs12885784	A	G
					52334225		rs114965032	G	A
					52334233		rs148884074	A	T
					52334242		rs17124680	A	C
					52334254		rs146988848	T	C
					52334263		rs1020889635	T	C

	Microhap	Chr	Molecular Extent (basepairs)	# SNPs	Nucleotide Position (GRCh37)		dbSNP rs-number	Alleles	
					52334281		rs568547747	C	A
					52334303		rs1022033223	G	A
63	mh14KK-048	14	194	8	74250537		rs1336460921	T	C
					74250553		rs149195448	G	A
					74250557		rs12717560	A	G
					74250562		rs76446474	G	A
					74250703		rs1437221210	C	G
					74250715		rs12878166	C	T
					74250724		rs1595304961	G	A
					74250730		rs12879393	T	C
64	mh15KK-067	15	196	7	46870730		rs79763993	T	C
					46870734		rs701463	T	G
					46870757		.	C	T
					46870771		rs1162241304	A	G
					46870829		rs7183984	T	C
					46870855		rs701464	C	T
					46870925		rs541910045	T	C
65	mh15KK-066	15	271	10	52484819		rs550005966	G	A
					52484831		rs147715323	T	C
					52484841		rs1077383	G	C
					52484871		rs183816954	G	T
					52484911		rs142502601	A	T
					52484950		rs1063902	A	C
					52484966		rs150251005	C	T
					52485024		rs4219	G	T
					52485049		rs948741853	G	T
					52485089		rs148957716	G	A
66	mh16KK-049	16	250	19	7209185		rs9937460	A	G
					7209200		rs184573741	A	T
					7209203		rs74009271	G	A
					7209208		rs9937467	A	C
					7209228		rs149818672	A	G
					7209240		.	T	C
					7209247		rs17670098	C	A

	Microhap	Chr	Molecular Extent (basepairs)	# SNPs	Nucleotide Position (GRCh37)		dbSNP rs-number	Alleles	
					7209248		rs113511754	A	C
					7209263		rs148562910	A	G
					7209296		rs973019410	T	C
					7209304		rs575286697	C	T
					7209311		rs12929083	A	G
					7209318		rs72765534	T	C
					7209328		.	A	T
					7209343		rs868244540	T	G
					7209370		rs115605200	C	T
					7209381		rs9926495	G	A
					7209428		rs72765535	A	G
					7209434		rs11642586	C	T,G
67	mh16KK-302	16	233	10	7587615		rs559824006	A	C
					7587676		rs1507022	G	T
					7587734		rs1395579	G	A
					7587746		rs1395580	C	T
					7587755		rs1395581	C	T
					7587771		rs377520498	C	T
					7587791		.	G	A
					7587804		rs1395582	T	A
					7587814		rs1297603692	A	T
					7587847		rs9939248	T	C
68	mh16KK-255	16	193	14	81970352		rs149471008	T	C
					81970353		rs16956011	G	A
					81970359		rs3934954	A	G
					81970366		rs3934955	A	C
					81970396		rs184092108	C	T
					81970407		rs3934956	C	T
					81970448		rs62044947	C	T,G
					81970449		rs1035277681	A	G
					81970453		.	G	T
					81970469		rs117206377	G	C
					81970471		rs3934957	C	G
					81970494		rs991027444	C	T
					81970495		rs4073828	A	G
					81970544		rs187444994	A	G

	Microhap	Chr	Molecular Extent (basepairs)	# SNPs	Nucleotide Position (GRCh37)		dbSNP rs-number	Alleles	
69	mh16KK-259	16	248	14	83973819		rs544552928	G	A
					83973836		rs143423041	C	G
					83973851		.	G	A
					83973881		rs140436204	G	C
					83973886		rs1597162269	A	G
					83973898		rs10163264	C	G
					83973918		rs10163267	G	C
					83973947		rs145717803	C	T
					83973956		rs10163270	G	C
					83973965		rs78983027	T	C
					83973984		rs544442291	T	C
					83974010		rs148944690	G	C
					83974058		rs934574266	G	A
					83974066		rs8058168	G	A
70	mh16KK-011	16	198	11	84285727		.	T	C
					84285738		rs74957131	C	T
					84285777		rs390112	T	C
					84285801		rs76460635	G	C
					84285825		rs188060999	C	T
					84285826		rs903080452	G	A
					84285828		rs390379	T	C
					84285833		rs426580	C	G
					84285849		rs422842	G	A
					84285862		rs7190826	C	A
					84285924		rs441199	G	A
71	mh16KK-262	16	258	13	87669318		rs193263518	G	T
					87669342		rs1186008027	C	T
					87669343		.	G	A
					87669371		rs825589	G	C
					87669385		rs1386620148	A	T
					87669386		rs1016861361	C	T
					87669387		rs1228760782	G	A
					87669407		rs825588	A	G
					87669535		rs863752	G	C
					87669545		rs861133	T	G
					87669562		rs79458371	C	T
					87669563		rs61069393	G	A

	Microhap	Chr	Molecular Extent (basepairs)	# SNPs	Nucleotide Position (GRCh37)		dbSNP rs-number	Alleles	
					87669575		rs118079046	C	T
72	mh17KK-272	17	260	11	52942335		rs531083518	T	G
					52942354		rs79327757	T	A
					52942414		rs111774039	C	A
					52942417		rs7207213	C	T
					52942421		rs2934896	A	G
					52942428		rs2934897	T	C
					52942456		rs7207239	C	T
					52942476		rs78871226	C	T
					52942491		rs16955257	C	A
					52942558		rs7212184	T	C
					52942594		rs944882339	T	C
73	mh17KK-012	17	245	13	77141265		rs56342141	A	G
					77141271		rs55714291	G	A
					77141277		rs56069218	A	G
					77141305		rs145799703	G	A
					77141308		rs7211029	C	T
					77141360		rs7211056	C	T
					77141394		rs76332411	A	G
					77141404		rs916022077	A	G
					77141407		rs55949744	G	A
					77141415		rs4789870	C	T
					77141433		rs982173999	G	A
					77141445		rs55877426	T	C
					77141509		.	T	C
74	mh17KK-013	17	245	10	77276404		rs72848020	C	T
					77276463		rs11651201	T	C
					77276472		.	C	T
					77276516		rs11651204	T	C
					77276517		rs116177500	G	A
					77276546		rs8074560	G	A
					77276591		rs1563446	G	T
					77276607		rs1297952819	G	A
					77276608		rs4465632	G	A
					77276648		rs147699838	C	T

	Microhap	Chr	Molecular Extent (basepairs)	# SNPs	Nucleotide Position (GRCh37)		dbSNP rs-number	Alleles	
75	mh17KK-278	17	187	7	78761546		rs4969266	C	T
					78761560		rs534028506	C	T
					78761607		rs11150747	C	T
					78761705		rs1202719973	A	G
					78761708		rs7225755	C	A
					78761726		rs72852775	A	G,T
					78761732		rs7224758	G	A
76	mh18KK-293	18	237	7	76089732		rs910492519	G	A
					76089844		rs80207925	C	G
					76089885		rs954593912	G	A
					76089886		rs621320	A	G
					76089907		rs621340	T	G
					76089945		rs678179	G	A
					76089968		rs621766	A	G
77	mh19KK-299	19	182	10	22729500		rs12985452	G	A
					22729505		rs1024001950	C	G
					22729548		.	G	A
					22729551		rs4932999	C	T
					22729552		rs979988710	G	A
					22729582		rs4932769	A	G
					22729613		rs2361019	T	A
					22729632		rs139451798	C	T
					22729653		rs2860462	G	A
					22729681		rs923762048	G	A
78	mh19KK-300	19	182	7	51451043		rs2411333	A	G
					51451046		rs10415306	C	T
					51451047		rs548501820	A	G
					51451087		rs34338963	A	C
					51451133		rs150844118	C	T
					51451134		rs139954003	G	A
					51451224		rs10415743	C	A
79	mh20KK-306	20	219	7	895313		rs370342593	C	T
					895325		rs11697918	A	G

	Microhap	Chr	Molecular Extent (basepairs)	# SNPs	Nucleotide Position (GRCh37)		dbSNP rs-number	Alleles	
					895418		rs533194	A	G
					895423		rs6086446	C	T
					895457		rs1014897	C	T,G
					895511		.	G	C
					895531		rs1014898	T	C
80	mh20KK-307	20	208	8	16513215		rs182552564	C	G
					16513236		rs1038592099	A	G
					16513260		rs6044080	T	C
					16513316		rs17674942	T	C,A
					16513342		rs6044081	A	G
					16513349		rs116146231	G	A
					16513400		rs16997830	A	C
					16513422		rs112240396	T	C
81	mh20KK-058	20	247	9	48844260		rs6122890	T	C
					48844293		rs6095836	A	G
					48844309		rs184999718	G	T
					48844348		rs6020381	G	T
					48844365		rs6012881	T	C
					48844389		rs4811008	T	G
					48844402		rs4811009	T	C
					48844460		rs541600111	A	G
					48844506		rs73121345	A	C
82	mh21KK-315	21	184	7	21880086		rs8126597	G	A
					21880100		rs76016088	G	A
					21880130		rs184686078	C	T
					21880140		rs1034293836	C	A
					21880191		rs8131148	C	T
					21880231		rs6517971	T	C
					21880269		rs111754000	C	T
83	mh21KK-316	21	255	7	27782968		rs961302	A	G
					27782992		rs17002090	C	T
					27783039		rs961301	A	G
					27783102		rs2830208	C	T
					27783184		rs73361625	G	A

	Microhap	Chr	Molecular Extent (basepairs)	# SNPs	Nucleotide Position (GRCh37)		dbSNP rs-number	Alleles	
					27783204		.	A	G
					27783222		.	A	G
84	mh21KK-318	21	235	10	41260129		rs370036702	A	G
					41260194		rs2250341	C	T
					41260227		rs149759443	T	C
					41260229		rs575743018	G	T
					41260234		rs2299752	C	T
					41260235		rs73904461	A	G,C
					41260260		rs17753847	C	T
					41260341		rs373008264	C	T
					41260343		rs796139151	A	C
					41260363		rs2299753	T	C
85	mh21KK-320	21	271	10	43062859		rs2838081	G	A
					43062881		rs1601636675	A	T
					43062912		rs558335378	T	C
					43062929		rs2838082	A	G
					43062959		rs533691353	T	C
					43063018		rs78902658	T	C
					43063044		rs2838083	A	G
					43063056		rs114395414	A	G
					43063086		rs370194397	C	T
					43063129		rs75885188	A	T
86	mh21KK-313	21	207	8	43942101		rs1051192053	C	A
					43942135		rs6586324	C	T
					43942151		rs539184331	C	G
					43942205		rs1373665440	G	C
					43942233		rs76960304	C	G
					43942249		rs6586325	G	T
					43942278		rs937203562	C	T
					43942307		rs6586326	T	C
87	mh21KK-324	21	179	9	46714536		rs542455866	C	T
					46714549		rs6518223	T	C
					46714586		rs7282557	G	A
					46714641		rs2838868	C	T,A

	Microhap	Chr	Molecular Extent (basepairs)	# SNPs	Nucleotide Position (GRCh37)		dbSNP rs-number	Alleles	
					46714650		rs932351589	C	T
					46714692		rs7279250	T	A
					46714707		rs8133697	G	A
					46714713		.	G	C
					46714714		rs80278660	C	T
88	mh22KK-328	22	244	7	18518651		rs1076115	T	C
					18518713		rs1040345053	C	T
					18518759		rs5746505	C	T
					18518783		rs13057610	T	C
					18518805		rs796553479	C	T
					18518829		rs4819661	G	A
					18518894		rs941511829	T	C
89	mh22KK-061	22	217	10	44763550		rs139200695	G	A
					44763606		rs763040	G	A
					44763631		rs1003170038	C	T
					44763651		rs5764924	A	G
					44763707		rs556260185	A	G,D
					44763713		rs574556415	T	C
					44763729		rs374119395	C	A
					44763751		.	C	G
					44763752		rs763041	A	G
					44763766		rs377313893	T	C
90	mh22KK-340	22	261	11	49060976		rs4925431	A	G
					49060987		rs4925399	A	G
					49060994		rs4925432	T	C
					49061016		rs4925400	A	G
					49061028		rs77899570	T	C
					49061044		rs4925401	G	A
					49061113		rs9628590	T	C
					49061126		rs117033898	C	T
					49061225		rs117448056	C	T
					49061226		rs369328449	C	T
					49061236		rs79558241	C	T

Note: For 15 microhaplotype alleles distributed among 8 microhaplotypes, deletions or insertions were present (in allele column: D=deletion, I=insertion) at very rare frequencies globally. One microhaplotype allele, occurring at low common frequencies, has a SNP deletion present in some populations in South Central Asia and Oceania; none of these populations were among the 16 sequenced via the mMHseq assay.

Table S2. Quality Control (QC) metrics

MiSeq sequencing run quality control metrics showing the average, median, minimum, and maximum values of various QC metrics for samples run in 6 independent sequencing runs.

	Yield (Mbases)	% passed filter (PF)	# Reads	% of raw clusters per lane	% perfect index reads	% One mismatch reads (index)	% of ≥ Q30 bases (PF)	Mean quality score (PF)
Average	185.1	100.0	737492.0	1.7	95.6	4.2	85.9	33.8
Median	177.0	100.0	706280.0	1.7	96.7	3.2	86.3	34.0
Min	59.0	100.0	234888.0	0.6	88.4	1.8	69.2	28.7
Max	547.0	100.0	2178628.0	5.1	98.2	11.3	92.3	35.8

**Quality control of sequenced reads**		
		Sample ID
Total number of samples sequenced	384	
**Read Count**		
Average Read Count per sample	705536	
Median Read Count per sample	685250	
Number of samples with fewer than 150,000 reads	8	S215, S216, S252, S253, S341, S342, S490, S509
**Amplicon Coverage**		
Average Amplicon Coverage at % >0.2X mean	79.96%	
Median Amplicon Coverage at % >0.2X mean	80.00%	
Number of amplicons with less than 2 SD of 0.2X mean	4	S254, S349, S350, S351
**Base Coverage**		
Average % of samples all with bases covered at ≥100 reads	94.20%	
Median % of samples all with bases covered at ≥100 reads	96.40%	
Number of samples with >75% of bases coverd at ≥100X	5	S252, S349, S350, S490, S509
Total number of samples that passed QC	381	
Total number of samples that failed QC	3	S252, S490, S509

Table S3. The top ranked 20 loci by average A_e in each of 6 world regions. Blue highlighting identifies the 6 best microhaplotypes in Table 4 to show how they rank in the 6 separate regions. There are 38 different microhaplotypes present in this table.

**Africa, Sub-Sahara**	**Avg Ae**	**N Africa, SW Asia, Europe**	**Avg Ae**	**South Central Asia**	**Avg Ae**	**East Asia**	**Avg Ae**	**Oceania**	**Avg Ae**	**Americas**	**Avg Ae**
16 populations		15 populations		24 populations		14 populations		7 populations		3 populations	
mh02KK-014	14.06	mh05KK-170	9.53	mh05KK-170	11.60	mh01KK-212	11.88	mh16KK-259	9.75	mh05KK-170	9.81
mh13KK-221	11.54	mh01KK-212	9.26	mh01KK-212	9.03	mh16KK-259	10.24	mh01KK-212	9.29	mh13KK-218	8.84
mh12KK-201	11.28	mh08KK-137	8.77	mh13KK-218	8.35	mh05KK-170	9.33	mh05KK-170	7.23	mh16KK-259	8.35
mh08KK-137	11.14	mh02KK-014	8.00	mh12KK-201	7.94	mh12KK-201	8.11	mh02KK-014	6.88	mh13KK-221	7.91
mh07KK-009	10.71	mh13KK-218	7.95	mh02KK-014	7.93	mh02KK-014	7.89	mh07KK-009	6.51	mh11KK-183	7.51
mh01KK-212	10.35	mh12KK-201	7.76	mh16KK-259	7.49	mh13KK-218	7.79	mh12KK-201	6.13	mh12KK-201	7.07
mh02KK-015	9.37	mh11KK-183	7.70	mh11KK-183	7.23	mh07KK-009	7.55	mh03KK-018	5.53	mh13KK-213	6.18
mh05KK-170	9.16	mh09KK-145	6.58	mh08KK-137	7.20	mh16KK-011	6.63	mh11KK-183	5.42	mh17KK-278	6.07
mh06KK-008	8.25	mh16KK-259	6.52	mh02KK-022	7.19	mh02KK-029	6.01	mh13KK-221	5.07	mh06KK-104	5.98
mh09KK-010	7.94	mh02KK-022	6.46	mh16KK-011	6.27	mh12KK-046	5.74	mh05KK-178	4.78	mh01KK-212	5.98
mh13KK-217	7.90	mh02KK-134	6.42	mh09KK-153	5.91	mh01KK-213	5.59	mh08KK-137	4.57	mh02KK-014	5.79
mh16KK-262	7.78	mh13KK-221	5.89	mh17KK-278	5.80	mh11KK-183	5.55	mh11KK-180	4.53	mh10KK-162	5.61
mh22KK-340	7.61	mh21KK-324	5.84	mh02KK-134	5.55	mh09KK-153	5.55	mh12KK-209	4.50	mh06KK-008	5.55
mh13KK-218	7.61	mh10KK-162	5.83	mh05KK-178	5.36	mh22KK-340	5.48	mh02KK-022	4.46	mh09KK-145	5.51
mh09KK-153	7.54	mh11KK-180	5.80	mh13KK-213	5.27	mh03KK-018	5.46	mh22KK-340	4.39	mh07KK-009	5.16
mh11KK-180	7.34	mh13KK-213	5.76	mh22KK-340	5.22	mh13KK-221	5.42	mh02KK-029	4.39	mh12KK-209	5.06
mh20KK-306	7.28	mh17KK-278	5.73	mh09KK-145	5.10	mh17KK-278	5.24	mh03KK-017	4.39	mh13KK-217	4.99
mh03KK-018	6.95	mh02KK-029	5.64	mh07KK-009	5.07	mh14KK-227	5.22	mh13KK-218	4.33	mh03KK-047	4.96
mh16KK-259	6.87	mh22KK-340	5.54	mh10KK-167	5.05	mh12KK-209	5.12	mh17KK-278	4.32	mh08KK-137	4.88
mh02KK-029	6.53	mh10KK-167	5.41	mh10KK-162	4.99	mh13KK-213	4.89	mh09KK-153	4.20	mh12KK-046	4.84

Figure S1. Quality control of 384 samples sequenced using mMHSeq Assay. The top panel shows the read depth per sample for all 384 samples. The samples with read depth coverage less than 150,000 reads are called out with sample name and their respective read counts. The mean read depth for all 384 samples is 705,536 reads. The bottom two panels show the uniformity of the amplicons within each sample (calculated and plotted as percentage of amplicons that are greater than 0.2X Mean amplicon coverage) and the percentage of the bases covered at greater than 100 reads per base. Samples that had less than 80% base coverage at 100 reads per base and those with amplicons at less than 2 Standard Deviations (SD) from the 0.2X mean were flagged. The Sample ID and their respective base coverage or percent amplicon greater than 0.2X mean are called out.

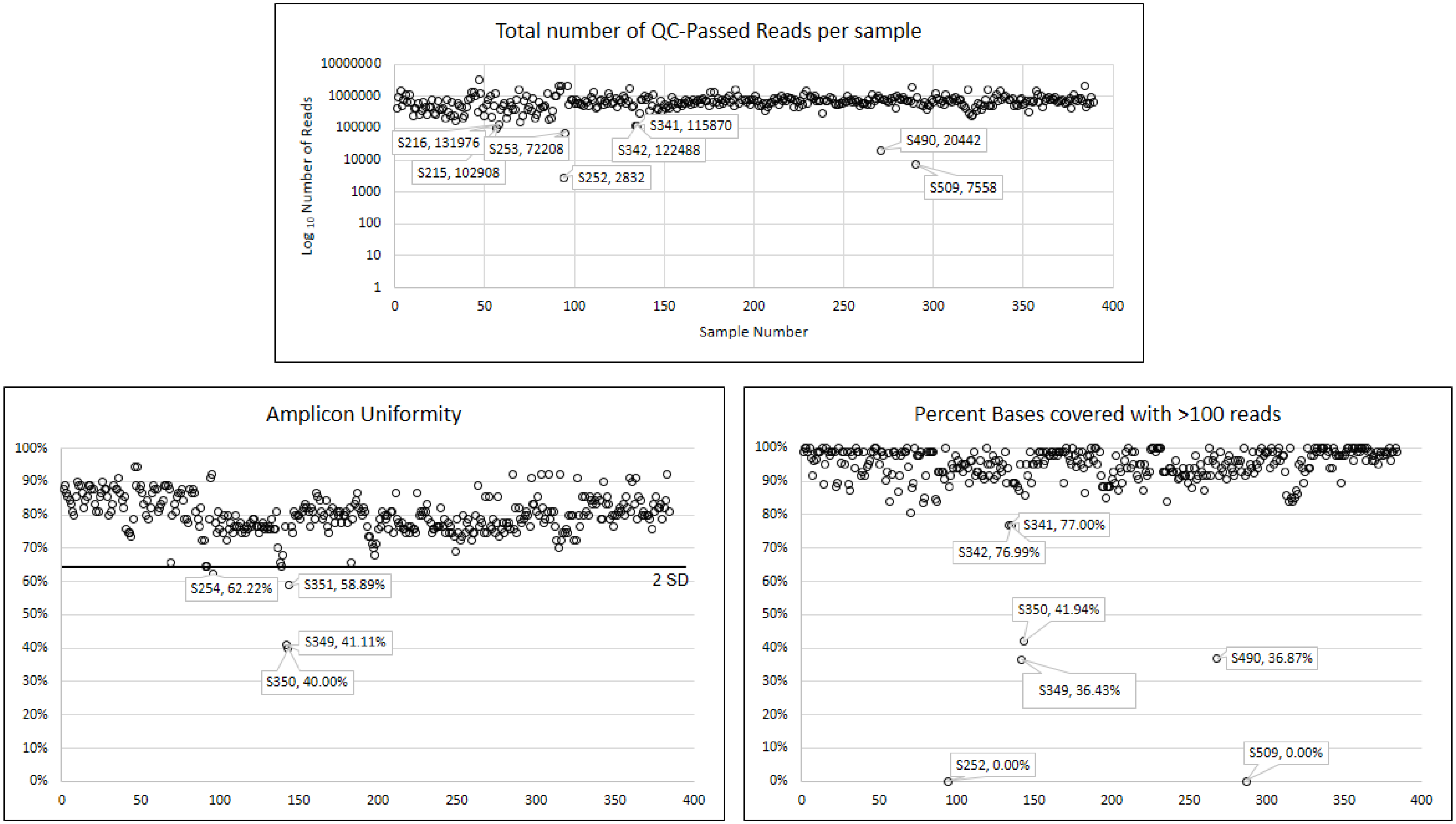

Figure S2. Distribution of average reads per genotype per individual for the 90 microhaplotype loci.

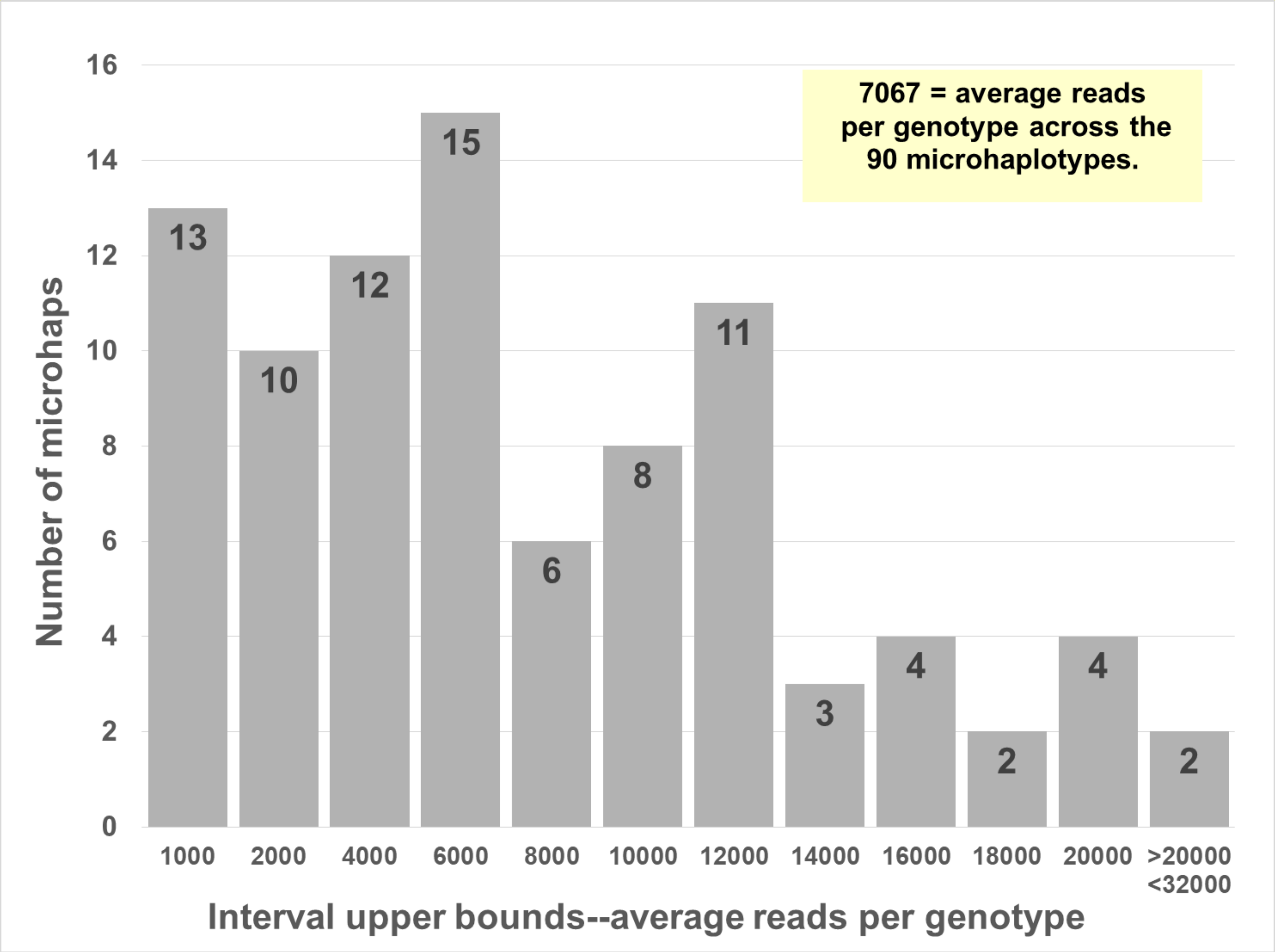

Figure S3. Average Ae in 79 populations for each of 90 microhaps.

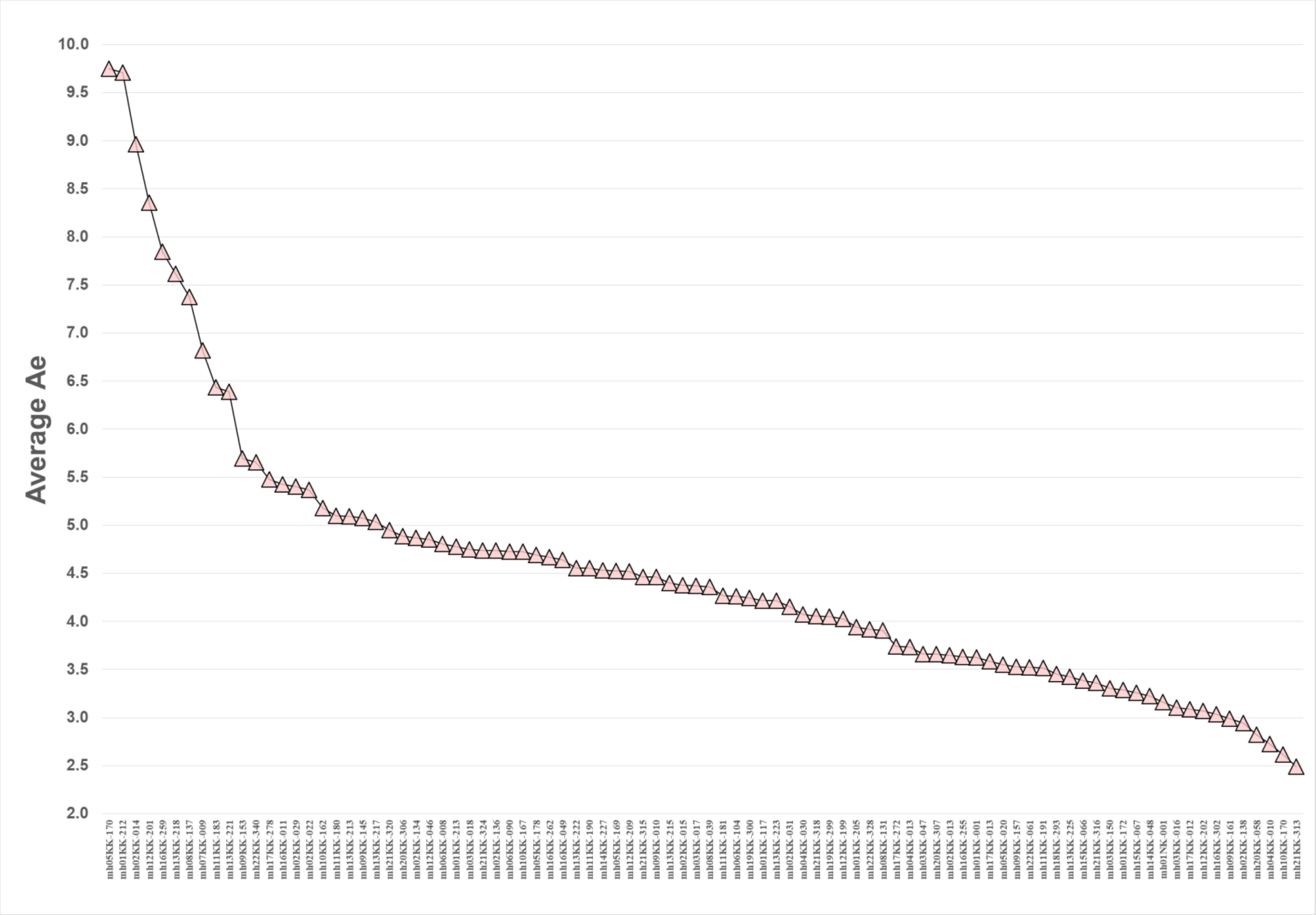

Figure S4. STRUCTURE individual bar plot at K=6 & 7 for all 79 populations

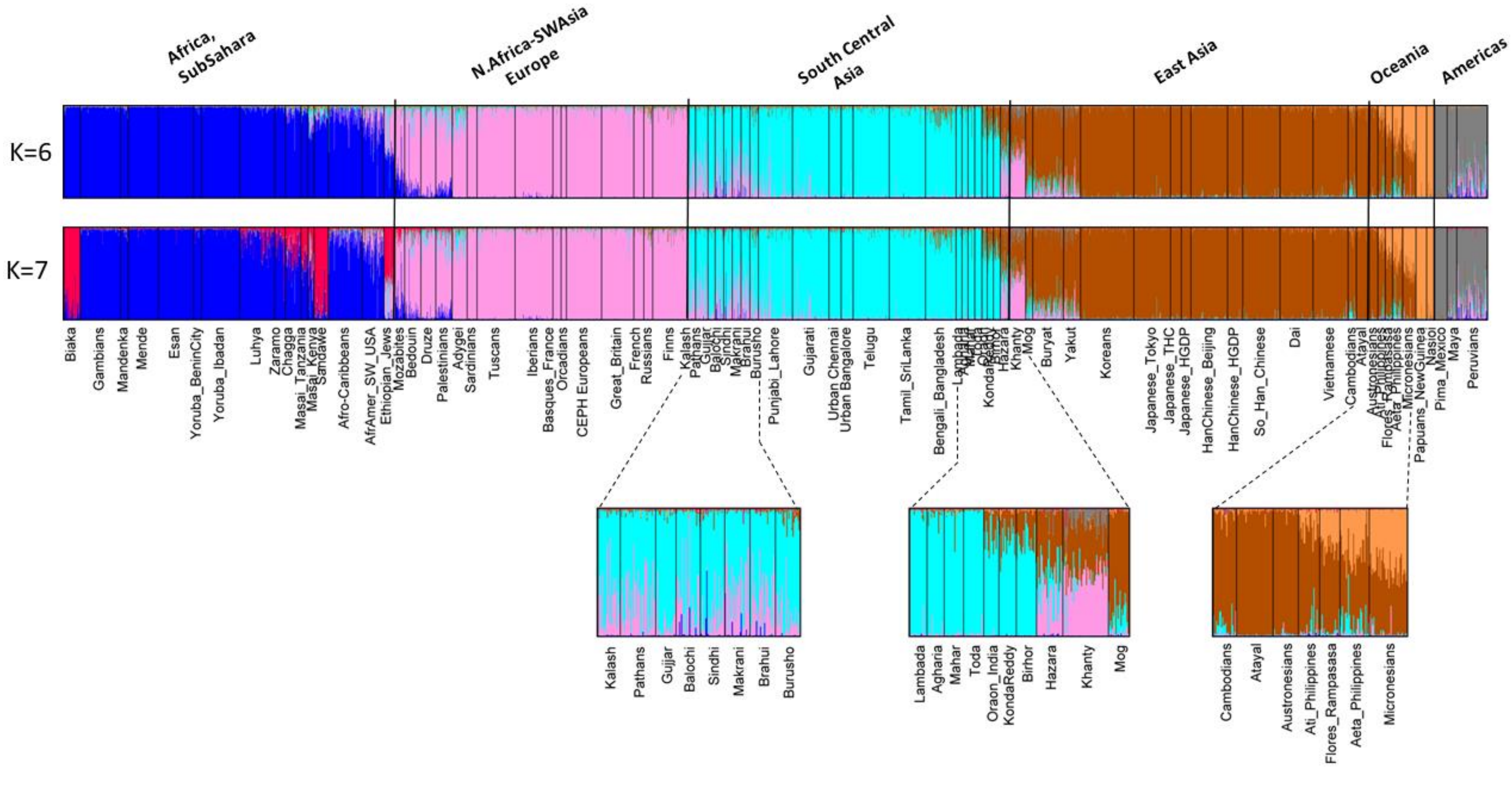

Figure S5. Likelihoods for STRUCTURE runs K=2 to 16 for 90 mh, 79 population dataset.

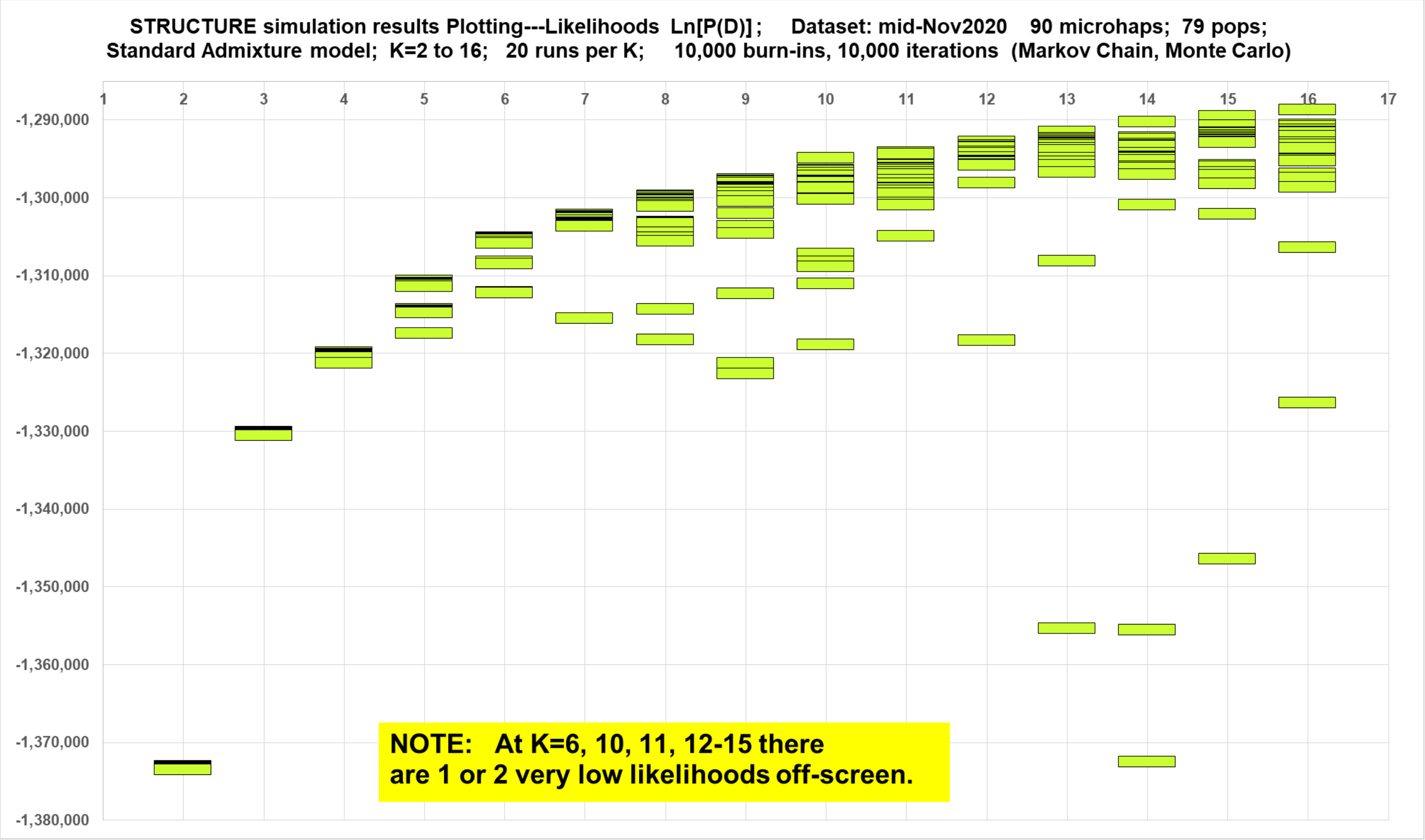

Figure S6. Individual bar plot of highest likelihood run at K=16 for the STRUCTURE analysis of 79 populations in the 90 microhaplotype dataset.

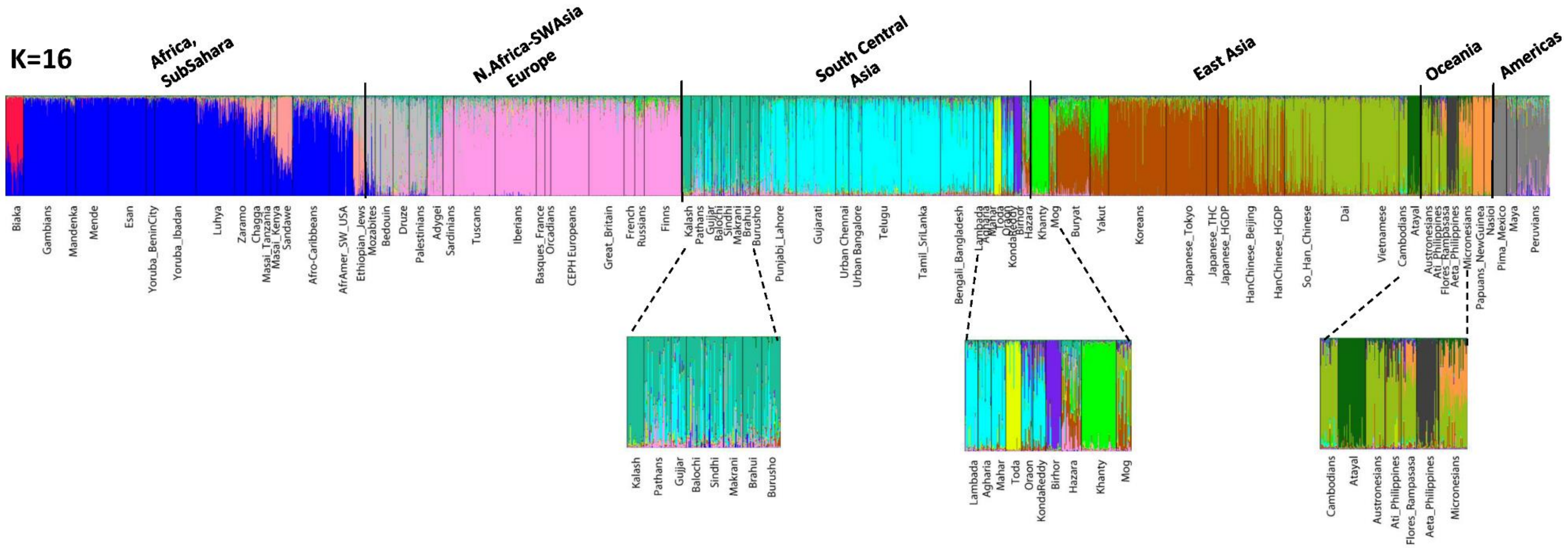

Figure S7. PCA of all 79 populations

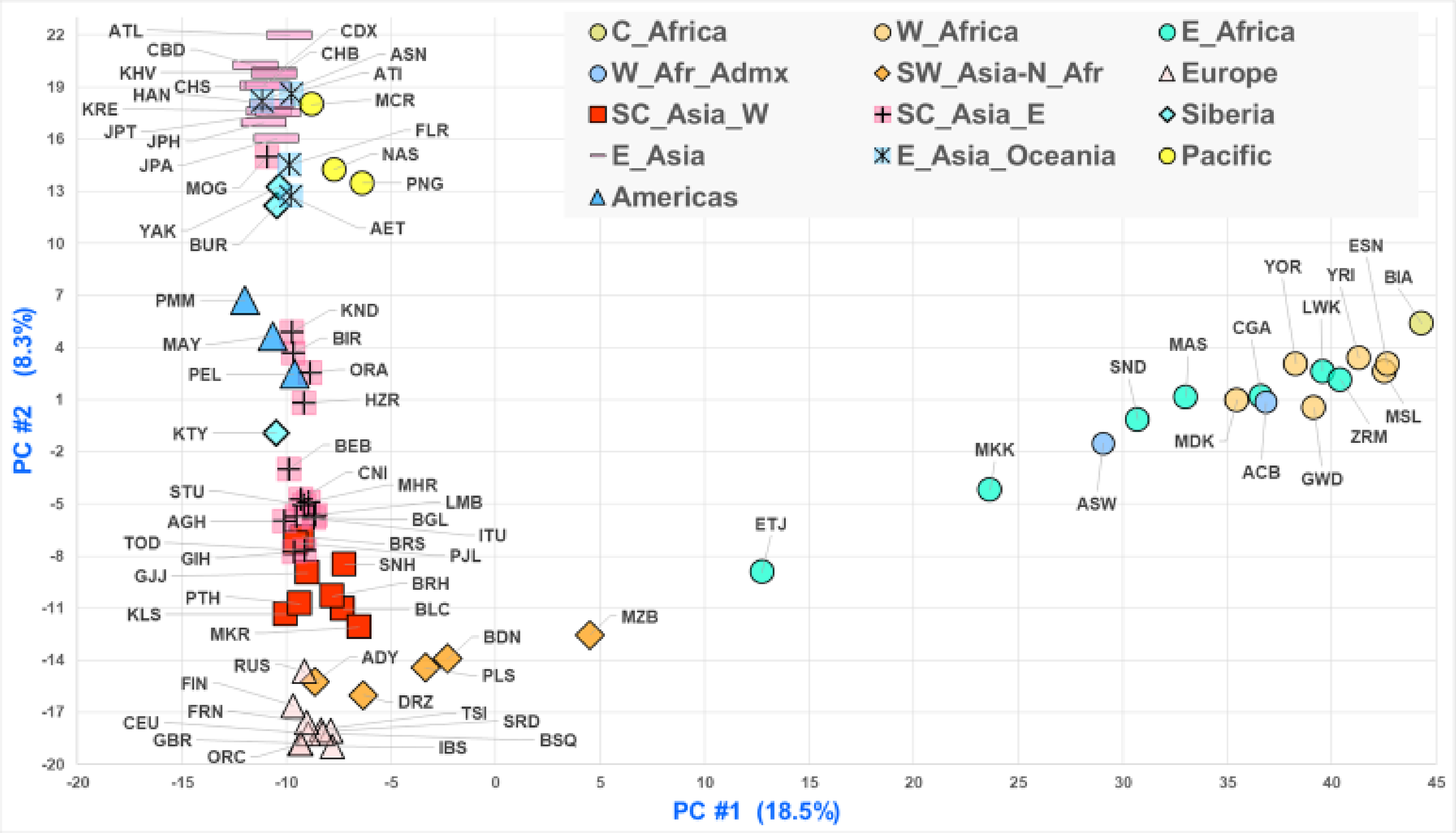

Figure S8. The best least squares (LS) population tree. A total of 294 different trees were evaluated

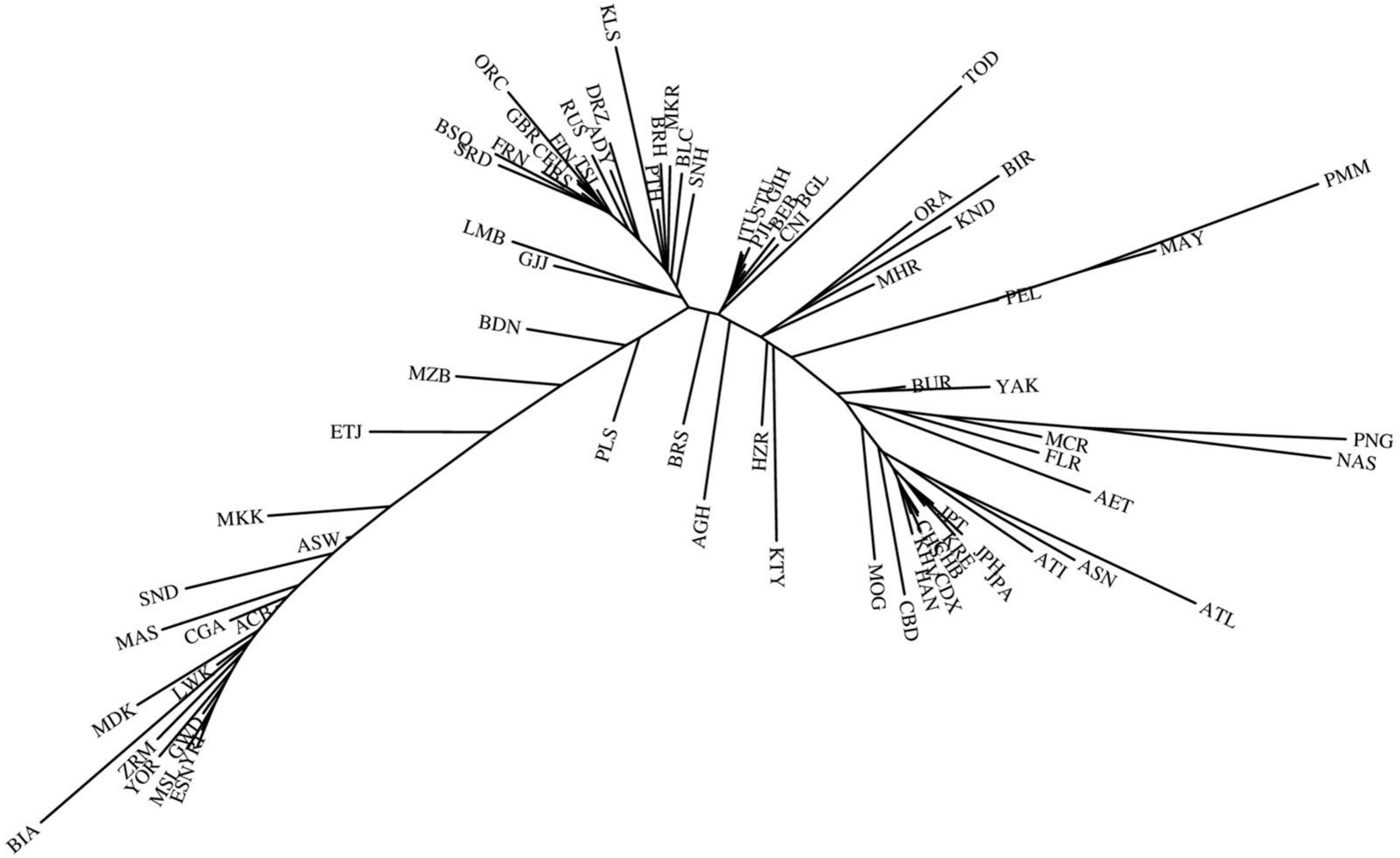

Figure S9a. PCA (PC1 x PC2) of the Africa – Southwest Asia subset of 21 populations

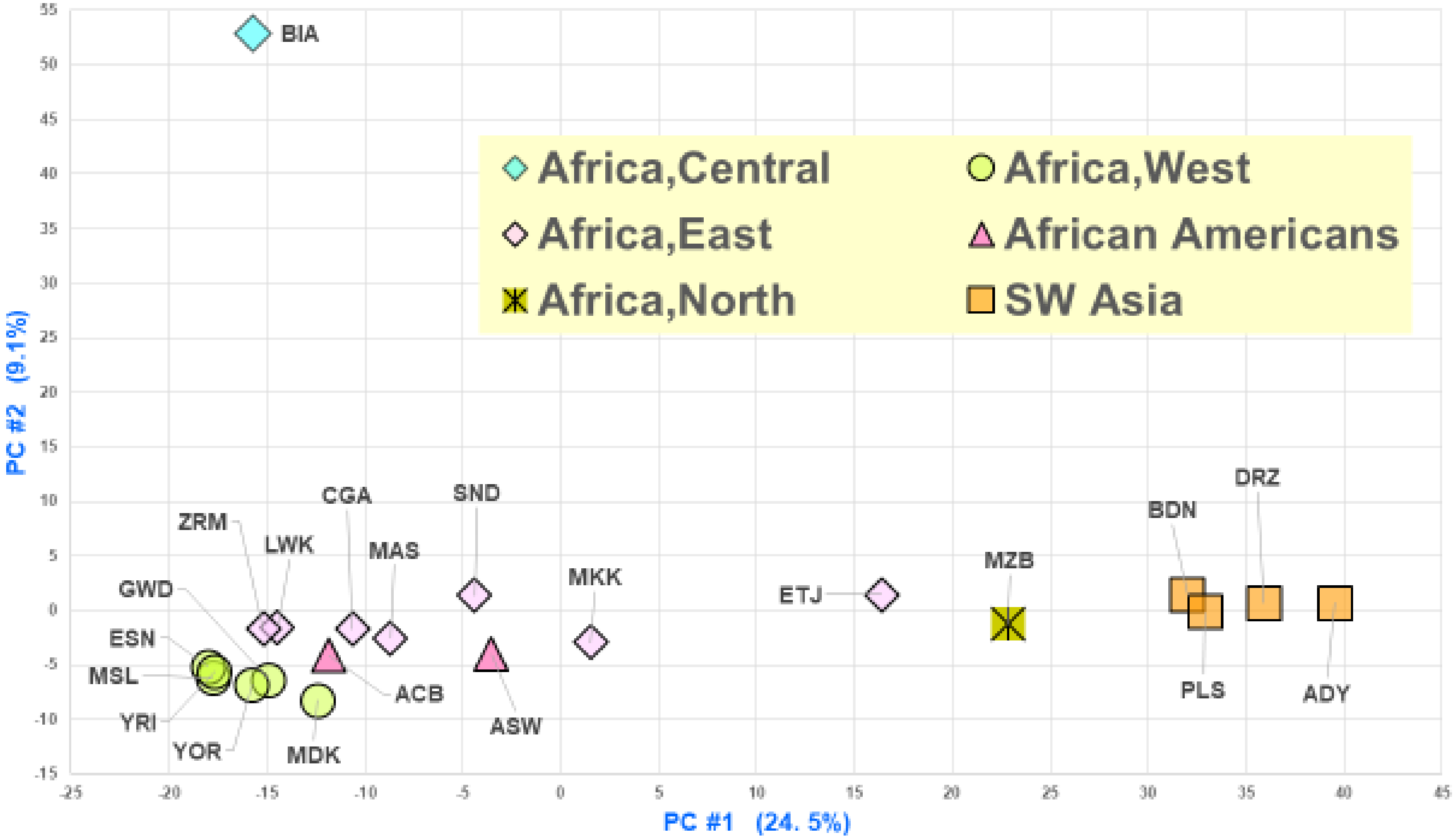

Figure S9b. PCA (PC1 x PC3) of the Africa – Southwest Asia subset of 21 populations

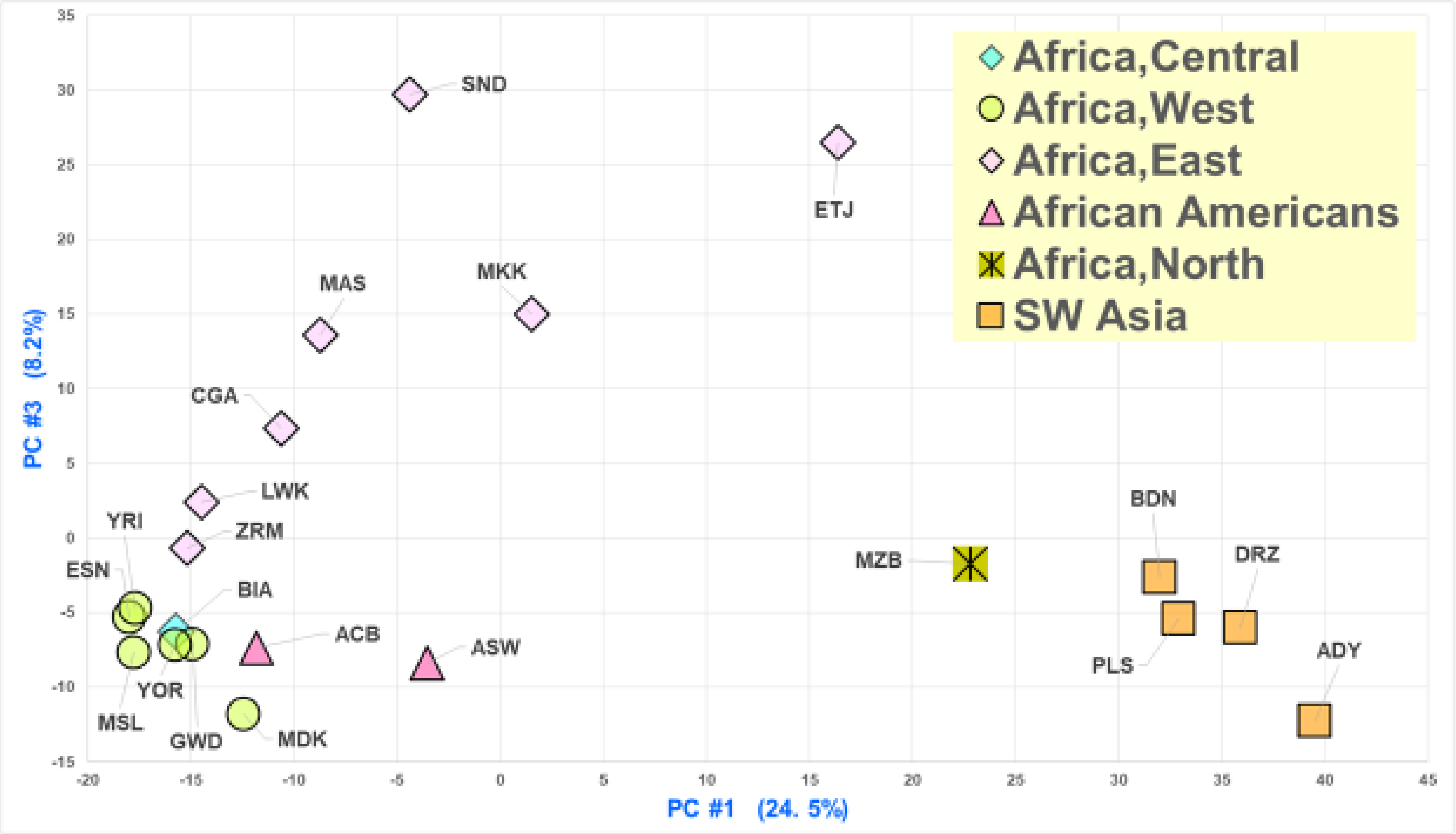

Figure S10. PCA of the Siberia – East Asia - Pacific subset of 21 populations

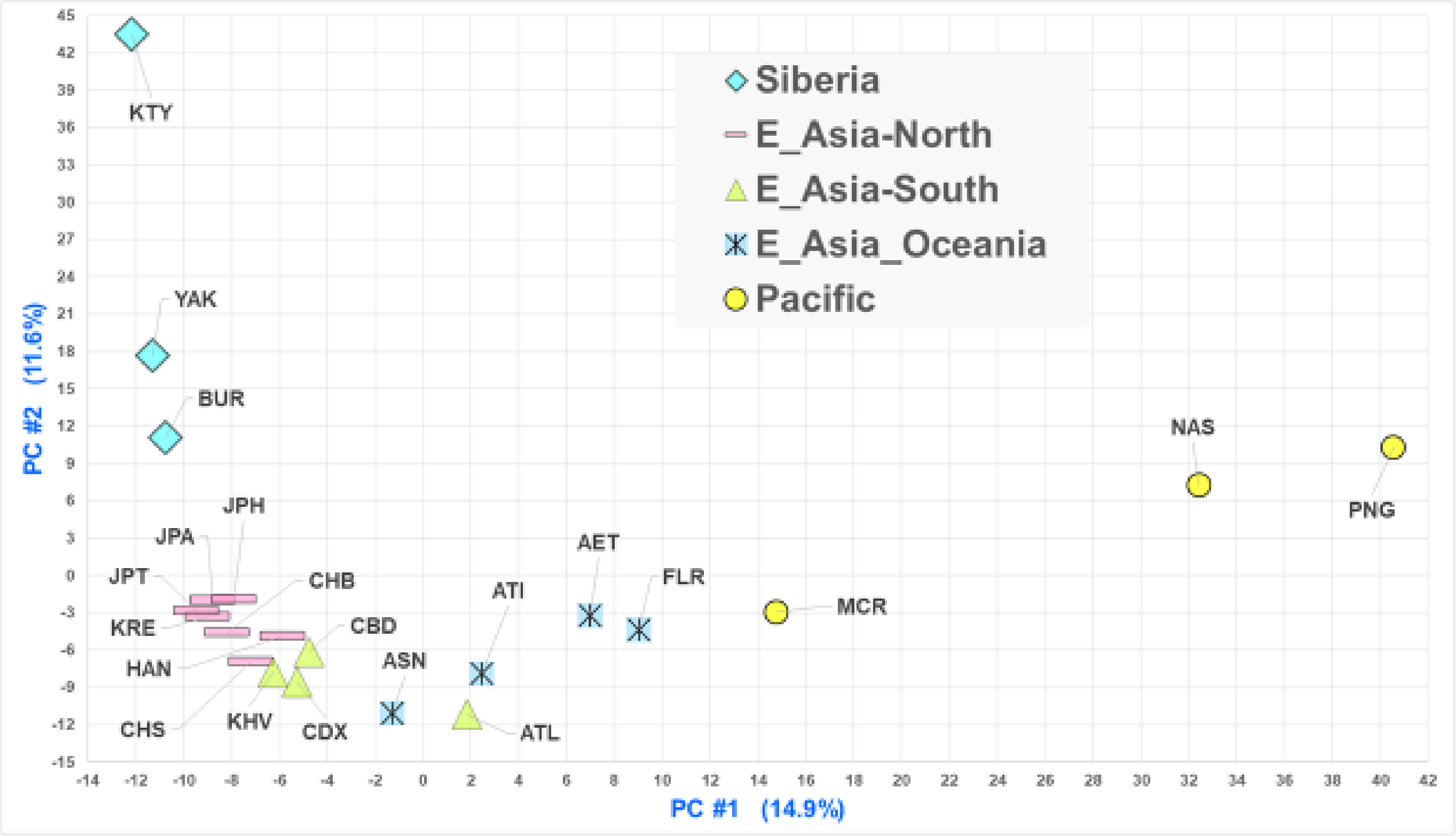

Supplement: Supplementary file 1 — Supplementary file1 (pdf 2762 KB) [file 439_2021_2382_MOESM1_ESM.pdf]
